# Mechanistic and Kinetic Insights into Cellular Uptake of Biomimetic Dinitrosyl Iron Complexes and Intracellular Delivery of NO for Activation of Cytoprotective HO-1

**DOI:** 10.1021/jacsau.4c00064

**Published:** 2024-03-29

**Authors:** Han Chiu, Anyelina Chau Fang, Yi-Hong Chen, Ru Xin Koi, Kai-Ching Yu, Li-Hung Hsieh, Yueh-Ming Shyu, Tarik Abdelkareem Mostafa Amer, Yi-Jen Hsueh, Yu-Ting Tsao, Yang-Jin Shen, Yun-Ming Wang, Hung-Chi Chen, Yu-Jen Lu, Chieh-Cheng Huang, Tsai-Te Lu

**Affiliations:** † Institute of Biomedical Engineering, National Tsing Hua University, Hsinchu 30013 Taiwan; ‡ Department of Biological Science and Technology, Institute of Molecular Medicine and Bioengineering, College of Biological Science and Technology, National Yang Ming Chiao Tung University, Hsinchu 300, Taiwan; § Department of Ophthalmology and Center for Tissue Engineering, Chang Gung Memorial Hospital, Taoyuan 33305, Taiwan; ∥ College of Medicine, Chang Gung University, Kwei-San, Taoyuan 33302, Taiwan; ⊥ Department of Neurosurgery, Chang Gung Memorial Hospital, Taoyuan 33305, Taiwan; # Department of Chemistry, National Tsing Hua University, Hsinchu 30013 Taiwan; ∇ Department of Chemistry, Chung Yuan Christian University, Taoyuan 32023, Taiwan

**Keywords:** nitric oxide, dinitrosyl iron complex, thiol-mediated uptake, heme oxygenase-1, regenerative medicine, mesenchymal stem cell, corneal endothelial cell

## Abstract

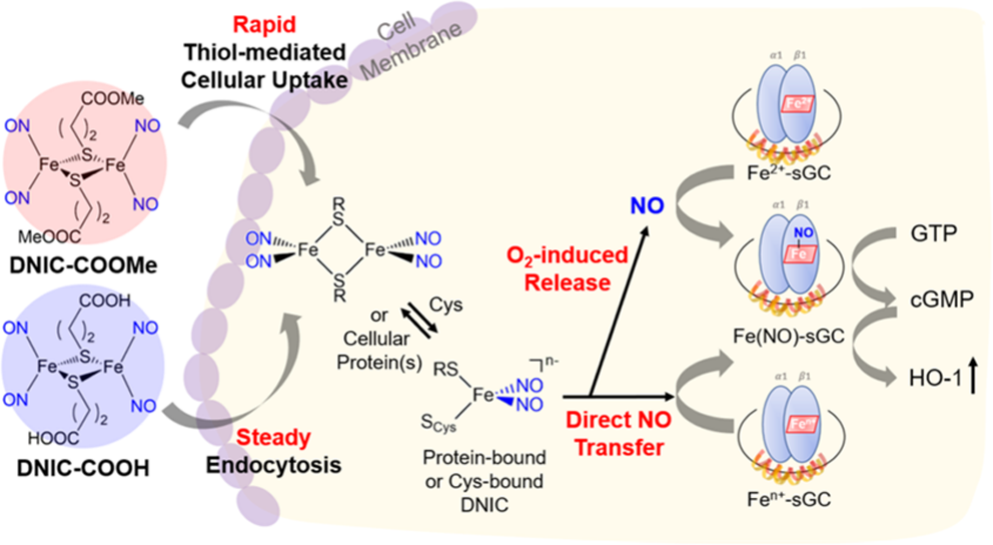

Dinitrosyl iron unit (DNIU), [Fe(NO)_2_], is a natural metallocofactor for biological storage, delivery, and metabolism of nitric oxide (NO). In the attempt to gain a biomimetic insight into the natural DNIU under biological system, in this study, synthetic dinitrosyl iron complexes (DNICs) [(NO)_2_Fe(μ-SCH_2_CH_2_COOH)_2_Fe(NO)_2_] (**DNIC–COOH**) and [(NO)_2_Fe(μ-SCH_2_CH_2_COOCH_3_)_2_Fe(NO)_2_] (**DNIC–COOMe**) were employed to investigate the structure–reactivity relationship of mechanism and kinetics for cellular uptake of DNICs, intracellular delivery of NO, and activation of cytoprotective heme oxygenase (HO)-1. After rapid cellular uptake of dinuclear **DNIC–COOMe** through a thiol-mediated pathway (*t*_max_ = 0.5 h), intracellular assembly of mononuclear DNIC [(NO)_2_Fe(SR)(S_Cys_)]^*n*−^/[(NO)_2_Fe(SR)(S_Cys-protein_)]^*n*−^ occurred, followed by O_2_-induced release of free NO (*t*_max_ = 1–2 h) or direct transfer of NO to soluble guanylate cyclase, which triggered the downstream HO-1. In contrast, steady kinetics for cellular uptake of **DNIC–COOH** via endocytosis (*t*_max_ = 2–8 h) and for intracellular release of NO (*t*_max_ = 4–6 h) reflected on the elevated activation of cytoprotective HO-1 (∼50–150-fold change at *t* = 3–10 h) and on the improved survival of **DNIC–COOH**-primed mesenchymal stem cell (MSC)/human corneal endothelial cell (HCEC) under stressed conditions. Consequently, this study unravels the bridging thiolate ligands in dinuclear **DNIC–COOH**/**DNIC–COOMe** as a switch to control the mechanism, kinetics, and efficacy for cellular uptake of DNICs, intracellular delivery of NO, and activation of cytoprotective HO-1, which poses an implication on enhanced survival of postengrafted MSC for advancing the MSC-based regenerative medicine.

## Introduction

Mesenchymal stem cell (MSC)-based transplantation therapies have been widely investigated for treating a variety of diseases/injuries that remain incurable nowadays.^1^ Although achieving enormous success in multiple animal and preclinical studies, a crucial impediment to realize the translational application of MSCs is the low survival rate of delivered cells at the desired site.^2−6^ The harsh microenvironment characterized by (a) inadequate oxygen and nutrient supply,^2,3^ (b) excessive pro-inflammatory cytokines,^7,8^ or (c) elevated oxidative stress significantly contributes to death of transplanted MSCs,^9,10^ thus compromising the ultimate therapeutic efficacy. In addition to MSC-based therapies, corneal transplantation is another cell-based therapy for sight-restoring in patients with corneal blindness.^11^ However, (a) inflammation due to surgical trauma or immunological rejection,^12^ (b) endothelial cell dysfunction,^13^ and (c) long-term loss of endothelial cell density (ECD) are formidable challenges resulting in corneal graft failure.^14−16^

Since the viability of administered cells is critical for the efficacy of MSC-based regenerative therapies and corneal transplantations, numerous approaches have been attempted to improve the survival of transplanted cells under a hostile milieu.^3,9,10,17−19^ Among the investigated methods, manipulation of heme oxygenase (HO)-1 has shown promising results in promoting the survival of MSC or protection of liver grafts/lung against the oxidative stress under myocardial/diabetic ischemia, ischemic stroke, and after liver/small bowel transplantation.^20−33^ Overexpression of HO-1 in MSC prior to transplantation by genetic engineering or pharmacological induction has been demonstrated to improve the postengrafted cell survival rate and thus enhance the resultant therapeutic benefits.^20−22^ Nevertheless, the safety issue remains a major concern for the clinical application of genetically manipulated cells,^24,34^ while the use of cobalt protoporphyrin, a potent inducer of HO-1, also raises concerns regarding metalloporphyrin-induced cytotoxicity or side effects.^35^ Therefore, strategies that can effectively upregulate HO-1 expression without compromising safety remain to be warranted to potentiate postengrafted MSC viability and therapeutic efficacy.

As a ubiquitous gasotransmitter, nitric oxide (NO) can activate the expression of HO-1 and promote cytoprotection toward vascular endothelium.^36−41^ Recently, biomimetic dinitrosyl iron complexes (DNICs) were explored as a prodrug for controlled delivery of NO and treatments of cancers,^42−44^ diabetic/bacteria-infected wound healing,^45−47^ hypertension,^48^ neurodegenerative disorders,^49−53^ and tissue engineering.^54−59^ In addition to the potential biomedical applications,^51^ systematic reviews on bioinorganic and coordination chemistry,^52,60,61^ synthetic methodology,^62,63^ and electronic structure study of biomimetic DNICs were also reported.^64^ As opposed to the other reported NO-delivery reagents, DNICs [(NO)_2_Fe(μ-SR)_2_Fe(NO)_2_] feature a unique O_2_-induced mechanism for the release of NO, of which the kinetics are modulated by the side chain of bridging thiolate ligands.^45,47,48,50,55,65^ In addition, rapid and reversible binding of DNIC [(NO)_2_Fe(μ-SCH_2_CH_2_OH)_2_Fe(NO)_2_] toward the protein-derived cysteine residues in gastrointestinal mucin and serum albumin occurs to promote the assembly of protein-bound DNIC [(NO)_2_Fe(SR)(S_Cys-protein_)]^*n*−^. Moreover, this protein-binding nature of DNIC enables the utilization of these endogenous protein vehicles for oral delivery of DNIC/NO into brain.^50^

**Scheme 1:**
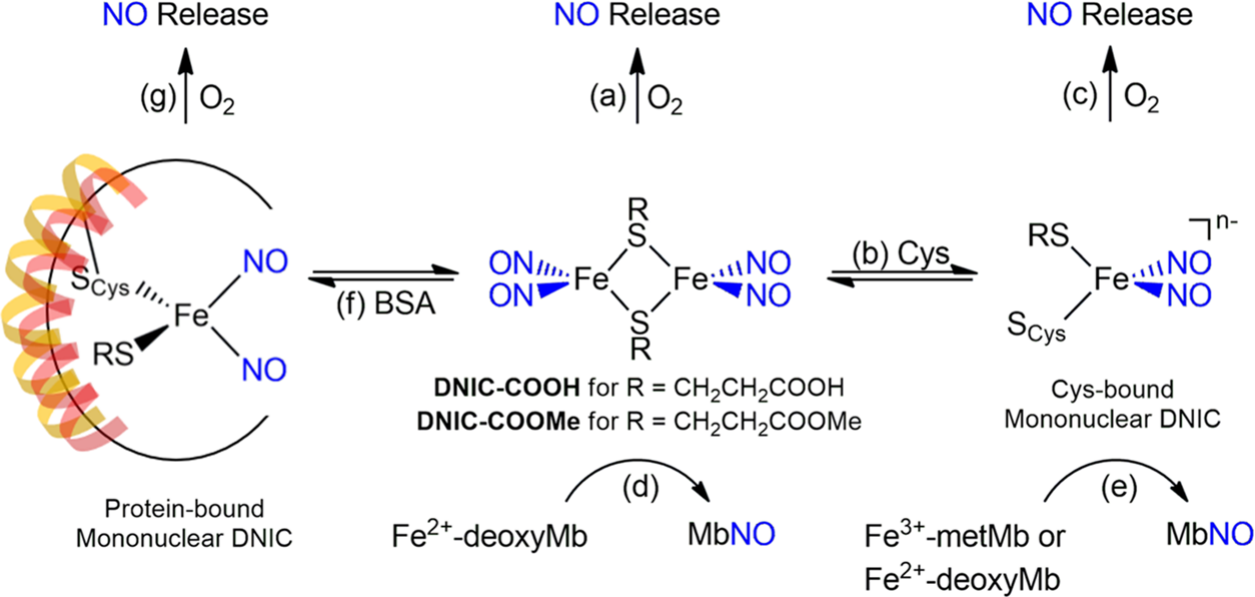
Mechanisms for Reversible Interactions between DNICs and Biological Thiols, Release of Free NO, and Direct Transfer of NO toward Fe^*n*+^-Porphyrin Center in Myoglobin

**Scheme 2:**
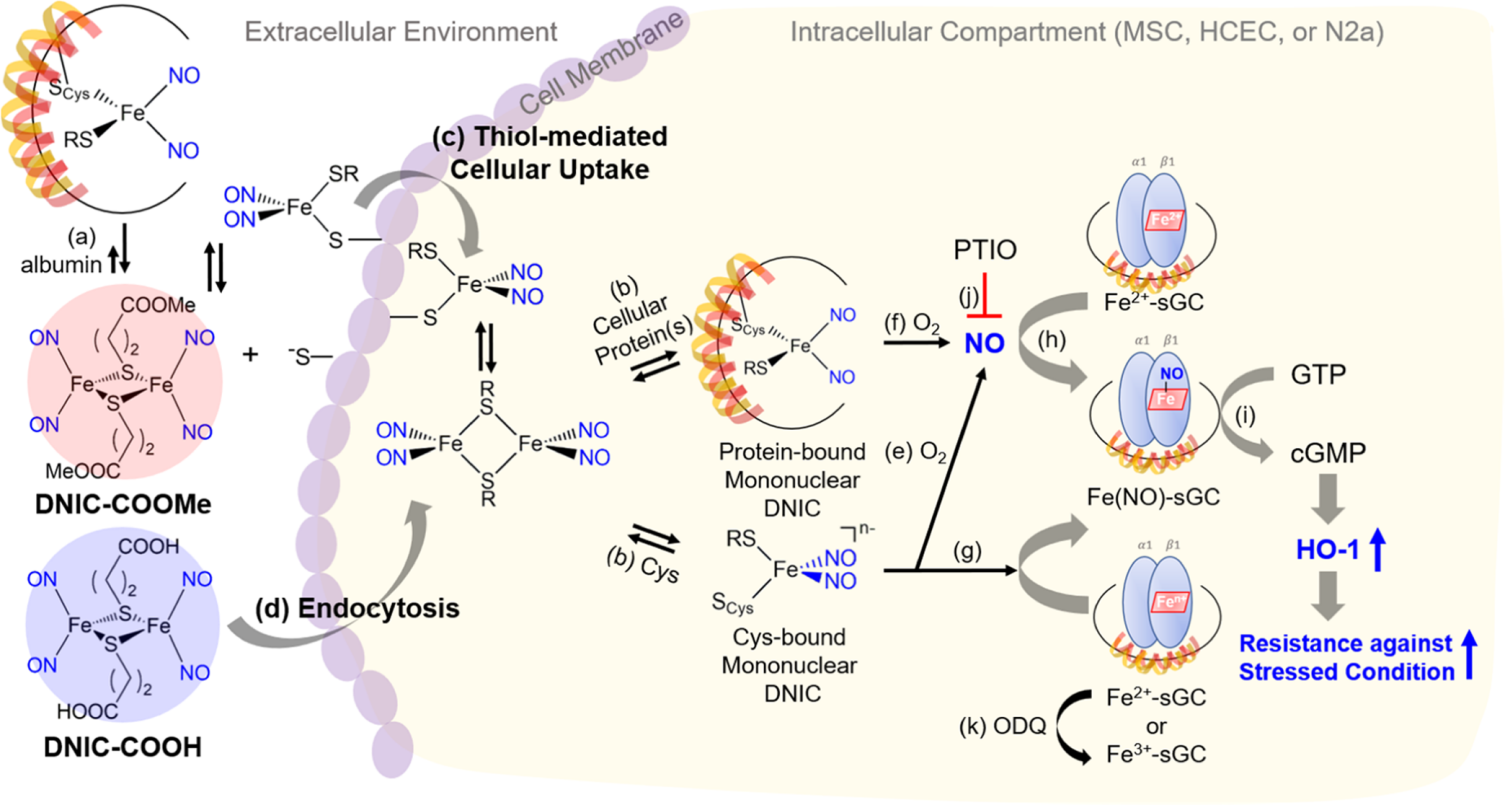
Schematic Illustration of the Reversible Interaction between DNICs and Extracellular Albumin, Cellular Uptake of DNICs via Alternative Mechanisms, Intracellular Transformation of DNICs and Delivery of NO, and Activation of Cytoprotective HO-1 for Enhanced Survival of MSC/HCEC under Stressed Conditions

Inspired by the potential of NO on potentiating MSC/corneal endothelial cell (CEC)-based transplantation therapy, herein, synthetic DNICs [(NO)_2_Fe(μ-SCH_2_CH_2_COOH)_2_Fe(NO)_2_] (**DNIC–COOH**) and [(NO)_2_Fe(μ-SCH_2_CH_2_COOCH_3_)_2_Fe(NO)_2_] (**DNIC–COOMe**) were employed to investigate the structure–reactivity relationship for kinetic and mechanistic control on cellular uptake of DNICs and intracellular delivery of NO (Schemes 1 and 2). As opposed to the slow binding of **DNIC–COOH** to the cysteine residue in bovine serum albumin (BSA) yielding BSA-bound DNIC, the fast BSA-binding kinetics of **DNIC–COOMe** may rationalize the rapid cellular uptake of **DNIC–COOMe** into MSC, human CEC (HCEC), and neuro-2a cells (N2a) through a thiol-mediated pathway. In comparison, **DNIC–COOH** displays a slow cellular uptake process through endocytosis instead of a thiol-mediated pathway, followed by steady intracellular release/transfer of NO. Of importance, this steady kinetics for intracellular delivery of NO featured by **DNIC–COOH** reflects on the extended/elevated overexpression of HO-1 and enhanced survival of **DNIC–COOH**-primed MSC/HCEC against hostile microenvironments.

## Results and Discussion

### Reversible Interactions between DNICs [(NO)_2_Fe(μ-SR)_2_Fe(NO)_2_] (R = CH_2_CH_2_COOH for DNIC–COOH, R = CH_2_CH_2_COOCH_3_ for DNIC–COOMe) and l-Cysteine (Cys) Regulate the Release of NO and Transfer of NO toward Fe^*n*+^-porphyrin Center (*n* = 2 or 3)

**Figure 1:**
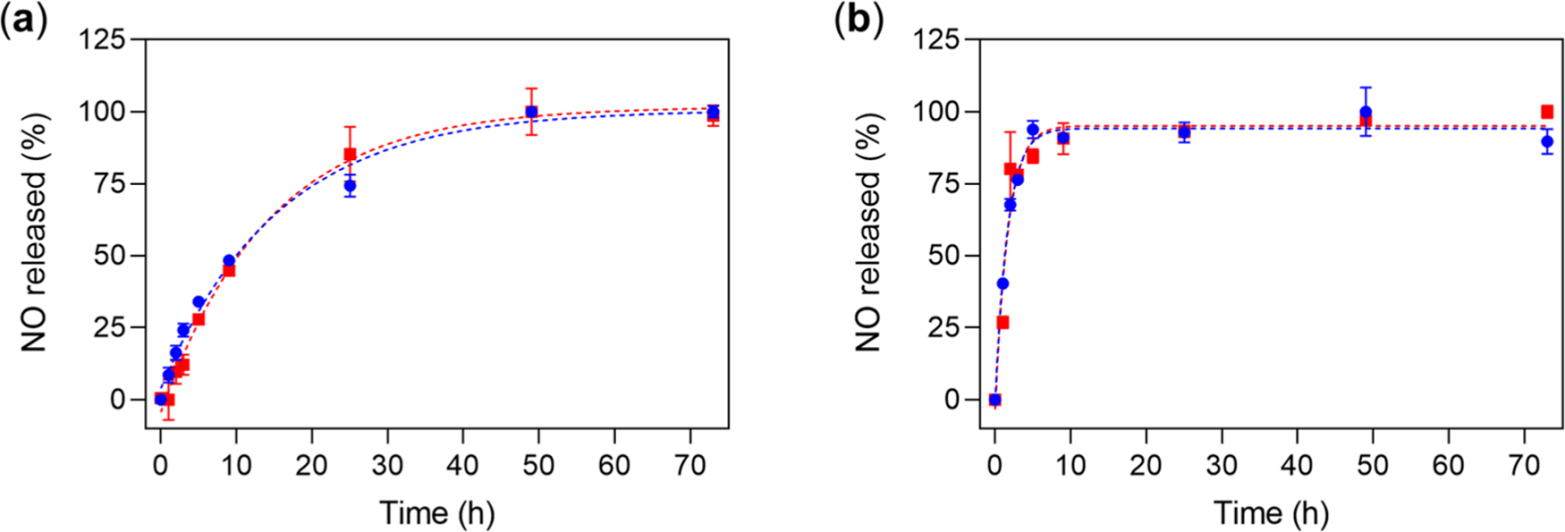
NO-release profiles of **DNIC–COOH** (25 μM, blue circle) and **DNIC–COOMe** (25 μM, red square) at pH 7.4 (a) without or (b) with the presence of 200 μM Cys under normoxia condition at 37 °C, which were fitted to pseudo-first-order kinetics (dashed line). Data show the mean ± standard deviation (SD) from three independent experiments.

As shown in Figure 1a and Scheme 1a, **DNIC–COOH** and **DNIC–COOMe** displayed a NO-release reactivity under normoxia condition, which is similar to the reported O_2_-induced release of NO from other dinuclear DNICs [(NO)_2_Fe(μ-SR)_2_Fe(NO)_2_].^45,47,48,50,55,65^ Based on the pseudo-first-order kinetic model, the *t*_1/2_ (or rate constant) for these processes investigated in this work and other published literature are summarized in Tables 1 and S1. During the O_2_-induced degradation of dinuclear **DNIC–COOH**/**DNIC–COOMe** in the presence of 2,4-di-*tert*-butyl phenol (DTBP), absent formation of 2,4-di-*tert*-butyl-6-nitrophenol (NO_2_-DTBP) and coupled bisphenol 3,3′,5,5′-tetra-*tert*-butyl-(1,1′-biphenyl)-2,2′-diol excluded the transient formation of peroxynitrite-derived ^•^OH/^•^NO_2_ species (Figure S1). Recently, a reversible interconversion between O_2_ and O_2_^–^ was discovered to be promoted by dinuclear DNICs [(NO)_2_Fe(μ-SEt)_2_Fe(NO)_2_]^*n*−^ (*n* = 0 and 1) without transient formation of ^•^OH/^•^NO_2_ species.^66^ As opposed to the generation of peroxynitrite and cytotoxic ^•^OH/^•^NO_2_ during the interaction between O_2_^–^ and NONOates (widely used chemical NO donors),^67^ this study unveiled the ^•^OH/^•^NO_2_-free NO-delivery reactivity and superoxide-scavenger nature of dinuclear DNICs [(NO)_2_Fe(μ-SR)_2_Fe(NO)_2_].

**Table 1:** Kinetics for NO-Delivery Reactivity and Degradation of DNICs under Alternative Conditions

NO-Delivery Reactivity of DNICs					
DNIC precursors	generated DNICs	media	additives	conditions	*t*_1/2_ or reaction time (h)
**DNIC–COOH**	**DNIC–COOH**^a^	phosphate buffer (pH 7.4)	**-**	normoxia	10.7 ± 1.4^e^
	[(NO)_2_Fe(SR)(S_Cys_)]^*n*−^b	phosphate buffer (pH 7.4)	Cys^a^	normoxia	1.2 ± 0.1^e^
	**DNIC–COOH**	phosphate buffer (pH 7.4)	deoxyMb^a^	anaerobic	-
	[(NO)_2_Fe(SR)(S_Cys_)]^*n*−^b	phosphate buffer (pH 7.4)	deoxyMb/Cys^a^	anaerobic	3.5^f^
	[(NO)_2_Fe(SR)(S_Cys_)]^*n*−^b	phosphate buffer (pH 7.4)	metMb/Cys^a^	anaerobic	4.0^f^
**DNIC–COOMe**	**DNIC–COOMe**^a^	phosphate buffer (pH 7.4)	**-**	normoxia	9.1 ± 0.6^e^
	[(NO)_2_Fe(SR)(S_Cys_)]^*n*−^b	phosphate buffer (pH 7.4)	Cys^a^	normoxia	1.2 ± 0.2^e^
	**DNIC–COOMe**	phosphate buffer (pH 7.4)	deoxyMb^a^	anaerobic	-
	[(NO)_2_Fe(SR)(S_Cys_)]^*n*−^b	phosphate buffer (pH 7.4)	deoxyMb/Cys^a^	anaerobic	2.0^f^
	[(NO)_2_Fe(SR)(S_Cys_)]^*n*−^b	phosphate buffer (pH 7.4)	metMb/Cys^a^	anaerobic	4.0^f^

Degradation of DNICs					
DNIC precursors	generated DNICs	media	additives	conditions	*t*_1/2_ (h)^g^
**DNIC–COOH**	[(NO)_2_Fe(SR)(S_Cys_)]^*n*−^b	phosphate buffer (pH 7.4)	Cys^a^	normoxia	0.7 ± 0.1
	[(NO)_2_Fe(SR)(S_Cys-albumin_)]^*n*−^c	αMEM	20% FBS	normoxia	4.8 ± 0.7
		MEM	5% FBS	normoxia	2.8 ± 0.5
		HSFM	2% FBS	normoxia	6.5 ± 2.0
	[(NO)_2_Fe(SR)(S_Cys_)]^n–^ or [(NO)_2_Fe(SR)(S_Cys-protein_)]^*n*−^d	MSC	-	normoxia	1.7 ± 0.4
		N2a	-	normoxia	2.1 ± 0.5
**DNIC–COOMe**	[(NO)_2_Fe(SR)(S_Cys_)]^*n*−^b	phosphate buffer (pH 7.4)	Cys^a^	normoxia	0.5 ± 0.1
	[(NO)_2_Fe(SR)(S_Cys-albumin_)]^*n*−^c	αMEM	20% FBS	normoxia	3.5 ± 0.5
		MEM	5% FBS	normoxia	1.8 ± 0.7
		HSFM	2% FBS	normoxia	1.5 ± 0.5
	[(NO)_2_Fe(SR)(S_Cys_)]^*n*−^ or [(NO)_2_Fe(SR)(S_Cys-protein_)]^*n*−^d	MSC	**-**	normoxia	1.0 ± 0.2
				hypoxia	1.8 ± 0.1
		N2a	**-**	normoxia	2.0 ± 0.5
				hypoxia	2.1 ± 0.4
		HCEC	**-**	normoxia	2.5 ± 0.9
				hypoxia	2.9 ± 0.2

a Concentrations of the reactants are **DNIC–COOH** = 25 μM, **DNIC–COOMe** = 25 μM, deoxyMb = 5 μM, and metMb = 5 μM. 200 μM of Cys was used for the study of NO-delivery reactivity of DNICs, while 10 mM of Cys was used for the study of degradation of DNICs.

b Obtained from the reaction of **DNIC–COOH**/**DNIC–COOMe** and Cys.

c Obtained from the reaction of **DNIC–COOH**/**DNIC–COOMe** and BSA in αMEM with 20% FBS, MEM with 5% FBS, or HSFM with 2% FBS.

d Obtained from treatments of **DNIC–COOH**/**DNIC–COOMe** to MSC, N2a, or HCEC.

e Half-life (*t*_1/2_) for release of NO from DNICs monitored using total nitrate/nitrite assay.

f Reaction time for complete conversion of deoxyMb/metMb into MbNO monitored using UV–vis spectroscopy.

g Half-life (*t*_1/2_) for degradation of DNICs monitored using EPR spectroscopy.

**Figure 2:**
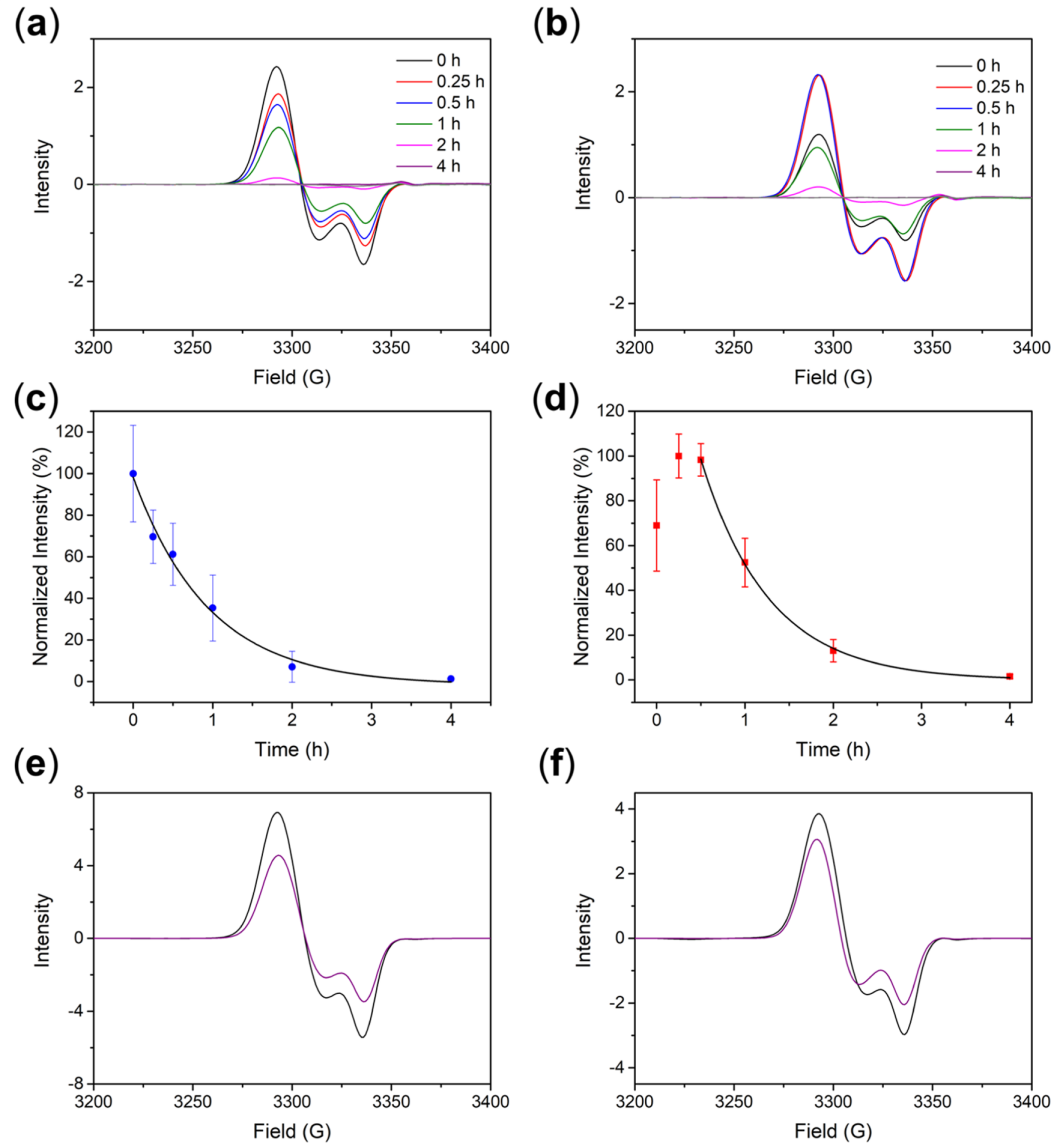
Time-dependent change of EPR spectra for (a) **DNIC–COOH** (50 μM) and (b) **DNIC–COOMe** (50 μM) at pH 7.4 with the presence of 10 mM Cys under normoxia condition. EPR spectra for **DNIC–COOH** and **DNIC–COOMe** (gray) are shown in the corresponding figures. Formation and decay of mononuclear DNIC [(NO)_2_Fe(SR)(S_Cys_)]^*n*−^ derived from the reaction of (c) **DNIC–COOH** (50 μM) and (d) **DNIC–COOMe** (50 μM) with 10 mM Cys at pH 7.4 under normoxia condition. Decays of mononuclear DNIC [(NO)_2_Fe(SR)(S_Cys_)]^*n*−^ under normoxia condition were fitted to pseudo-first-order kinetics (black line). Data show the mean ± SD from three independent experiments. EPR spectra for (e) **DNIC–COOH** (50 μM) and (f) **DNIC–COOMe** (50 μM) with 10 mM Cys incubated under anaerobic condition for 0 h (black) and 4 h (purple), respectively.

Inspired by the reported interactions between dinuclear DNICs [(NO)_2_Fe(μ-SR)_2_Fe(NO)_2_] and R′SH/[SR′]^−^ leading to the assembly of EPR-active mononuclear {Fe(NO)_2_}^9^ DNICs [(NO)_2_Fe(SR)(SR′)]^−^,^50,55,68,69^ reactions of **DNIC–COOH**/**DNIC–COOMe** with biological thiols, i.e., l-cysteine (Cys) or serum albumin (BSA), were further explored. As shown in Figure 2a,b, treatment of 10 mM of Cys to **DNIC–COOH** (or **DNIC–COOMe**) resulted in the formation of a distinctive EPR signal at *g* = 2.041, 2.035, and 2.015 (or *g* = 2.041, 2.035, and 2.014, Tables 2 and S2). Similar to the reactions between DNICs and Cys reported in the literature, these EPR investigations supported Cys-induced conversion of **DNIC–COOH**/**DNIC–COOMe** into the proposed mononuclear {Fe(NO)_2_}^9^ DNICs [(NO)_2_Fe(SR)(S_Cys_)]^*n*−^ (Scheme 1b). Moreover, complete degradations of mononuclear DNICs [(NO)_2_Fe(SR)(S_Cys_)]^*n*−^ were observed after incubation for 4 h based on the time-dependent decrease of distinctive EPR signals at *g*_av_ = 2.03 under normoxia condition (Figure 2a–d). In contrast, mononuclear DNICs [(NO)_2_Fe(SR)(S_Cys_)]^*n*−^ displayed an enhanced stability under anaerobic condition (Figure 2e,f), which suggested an O_2_-induced mechanism for decomposition of mononuclear DNICs [(NO)_2_Fe(SR)(S_Cys_)]^*n*−^ (Scheme 1c). Using a pseudo-first-order kinetic model, kinetics for O_2_-induced decomposition of mononuclear DNICs [(NO)_2_Fe(SR)(S_Cys_)]^*n*−^ were determined as *t*_1/2_ = 0.7 ± 0.1 h for SR = SCH_2_CH_2_COOH and *t*_1/2_ = 0.5 ± 0.1 h for SR = SCH_2_CH_2_COOMe (Figure 2c,d). In comparison with the kinetics for release of NO from **DNIC–COOH**/**DNIC–COOMe** without/with the presence of 200 μM of Cys (Figure 1 and Table 1), Cys accelerates the O_2_-induced decomposition of dinuclear **DNIC–COOH**/**DNIC–COOMe** and the release of NO through formation of mononuclear DNICs [(NO)_2_Fe(SR)(S_Cys_)]^*n*−^.

**Table 2:** EPR Parameters for Mononuclear and Dinuclear DNICs Explored in This Study

DNIC precursors	generated DNICs	additives	media	EPR parameters (*g*_1_, *g*_2_, *g*_3_)
**DNIC–COOH**	**DNIC–COOH**	-	PBS (pH 7.4)	EPR silent
	**DNIC–COOH**	-	αMEM	EPR silent
	**DNIC–COOH**	-	MEM	EPR silent
	**DNIC–COOH**	-	HSFM	EPR silent
	**DNIC–COOH**	20% NEM-treated FBS	αMEM	EPR silent
	**DNIC–COOH**	5% NEM-treated FBS	MEM	EPR silent
	**DNIC–COOH**	2% NEM-treated FBS	HSFM	EPR silent
	[(NO)_2_Fe(SR)(S_Cys_)]^*n*−^a	Cys	PBS (pH 7.4)	(2.041, 2.035, 2.015)
	[(NO)_2_Fe(SR)(S_Cys-albumin_)]^*n*−^b	20% FBS	αMEM	(2.044, 2.037, 2.015)
		5% FBS	MEM	(2.042, 2.036, 2.015)
		2% FBS	HSFM	(2.042, 2.035, 2.014)
	[(NO)_2_Fe(SR)(S_Cys_)]^*n*−^ or [(NO)_2_Fe(SR)(S_Cys-protein_)]^*n*−^c	-	MSC	(2.041, 2.034, 2.015)
		-	N2a	(2.041, 2.034, 2.014)
		-	HCEC	(2.040, 2.034, 2.015)
**DNIC–COOMe**	**DNIC–COOMe**	-	PBS (pH 7.4)	-
	**DNIC–COOMe**	-	αMEM	EPR silent
	**DNIC–COOMe**	-	MEM	EPR silent
	**DNIC–COOMe**	-	HSFM	EPR silent
	**DNIC–COOMe**	20% NEM-treated FBS	αMEM	EPR silent
	**DNIC–COOMe**	5% NEM-treated FBS	MEM	EPR silent
	**DNIC–COOMe**	2% NEM-treated FBS	HSFM	EPR silent
	[(NO)_2_Fe(SR)(S_Cys_)]^*n*−^a	Cys	PBS (pH 7.4)	(2.041, 2.035, 2.014)
	[(NO)_2_Fe(SR)(S_Cys-albumin_)]^*n*−^b	20% FBS	αMEM	(2.043, 2.036, 2.015)
		5% FBS	MEM	(2.042, 2.036, 2.015)
		2% FBS	HSFM	(2.042, 2.035, 2.014)
	[(NO)_2_Fe(SR)(S_Cys_)]^n–^ or [(NO)_2_Fe(SR)(SCys-protein)]^*n*−^c	-	MSC	(2.041, 2.035, 2.015)
		-	N2a	(2.041, 2.034, 2.014)
		-	HCEC	(2.040, 2.034, 2.014)

a Obtained from the reaction of **DNIC–COOH**/**DNIC–COOMe** and Cys.

b Obtained from the reaction of **DNIC–COOH**/**DNIC–COOMe** and BSA in αMEM with 20% FBS, MEM with 5% FBS, or HSFM with 2% FBS.

c Obtained from treatments of **DNIC–COOH**/**DNIC–COOMe** to MSC, N2a, or HCEC.

**Figure 3:**
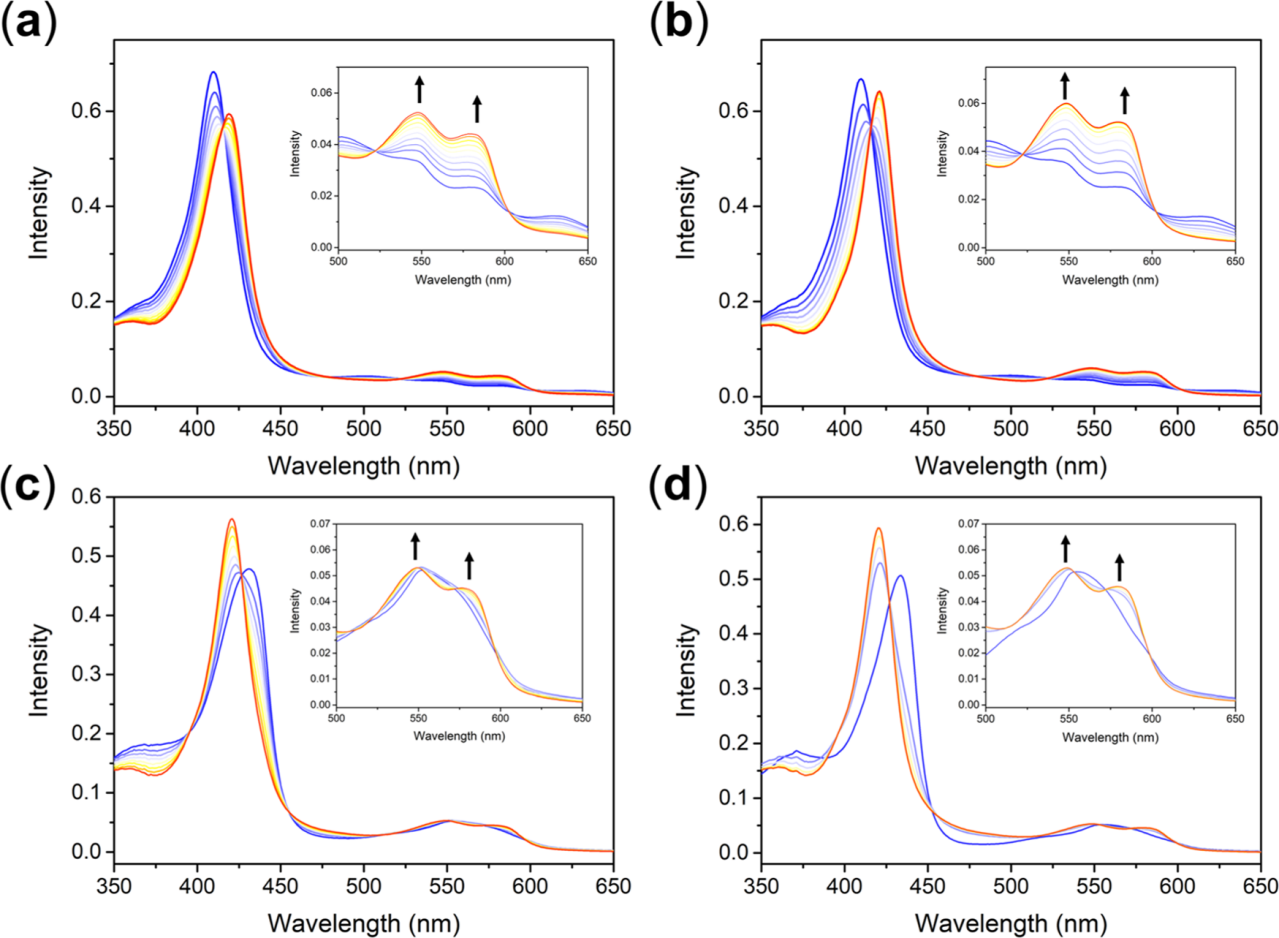
Reactions of metMb (5 μM) with (a) **DNIC–COOH** (5 μM) and Cys (200 μM) and (b) **DNIC–COOMe** (5 μM) and Cys (200 μM), respectively, under anaerobic condition monitored by UV–vis spectroscopy. Reactions of deoxyMb (5 μM) with (c) **DNIC–COOH** (5 μM) and Cys (200 μM) and (d) **DNIC–COOMe** (5 μM) and Cys (200 μM), respectively, under anaerobic condition monitored by UV–vis spectroscopy.

Besides the O_2_-induced release of free NO from dinuclear **DNIC–COOH**/**DNIC–COOMe** and mononuclear DNICs [(NO)_2_Fe(SR)(S_Cys_)]^*n*−^, interactions of DNICs with Fe^2+^-/Fe^3+^-porphyrin center in deoxy-/met-myoglobin (deoxyMb/metMb) for direct transfer of NO/NO^–^ yielding nitrosylated myoglobin (MbNO) were further explored under anaerobic condition.^55,65,70^ Recently, dinuclear **DNIC–COOH** was reported to be inert toward the Fe^3+^-porphyrin center in metMb, which is in contrast to efficient nitrosylation of the Fe^2+^-porphyrin center in deoxyMb by dinuclear **DNIC–COOH** leading to generation of MbNO (Scheme 1d).^53^ As shown in Figure 3a,b, treatment of 200 μM of Cys to the reaction solution of 5 μM of metMb and **DNIC–COOH** (or **DNIC–COOMe**), of interest, resulted in the shift of UV–vis absorption bands from (410, 502, 629) nm to (419, 548, 578) nm, which indicated the formation of MbNO. Regarding the absent reaction between metMb and Cys (Figure S2), Cys-induced conversion of dinuclear **DNIC–COOH**/**DNIC–COOMe** into mononuclear DNIC [(NO)_2_Fe(SR)(S_Cys_)]^*n*−^ may occur, followed by direct transfer of NO^–^ to the Fe^3+^-porphyrin center in metMb yielding MbNO (Scheme 1b,e). In the reported study of NO^–^-transfer reactions between DNICs and metMb, the inert nature of dinuclear DNIC [(NO)_2_Fe(μ-SEt)_2_Fe(NO)_2_] toward metMb was ascribed to its ^•^NO-delivery nature.^70,71^ That is, based on the valence-to-core X-ray emission spectroscopy, the electronic structure of NO ligands in the dinuclear DNIC [(NO)_2_Fe(μ-SEt)_2_Fe(NO)_2_] was determined as −0.15 ± 0.18, namely, a ^•^NO-delivery nature. In comparison, mononuclear DNIC [(NO)_2_Fe(SPh)_2_]^−^ features an enhanced NO^–^ character (electronic structure of Fe-bound NO = −0.77 ± 0.18) and an NO^–^-transfer reactivity toward metMb.^70,71^ Presumably, the enhanced NO^–^ character and NO^–^-transfer reactivity of mononuclear DNIC [(NO)_2_Fe(SPh)_2_]^−^ may project on the mechanisms for transformation of metMb into MbNO promoted by mononuclear DNIC [(NO)_2_Fe(SR)(S_Cys_)]^*n*−^ derived from reaction of dinuclear **DNIC–COOH**/**DNIC–COOMe** and Cys. In addition to the transformation of metMb into MbNO, mononuclear DNICs [(NO)_2_Fe(SR)(S_Cys_)]^*n*−^ also exhibited nitrosylation reactivity for conversion of deoxyMb into MbNO (Figure 3c,d, Table 1, Scheme 1e).

Based on the investigations discussed above and reported in the literature, potential mechanisms and kinetics for NO-delivery reactivity of DNICs are summarized in Scheme 1 and Table 1. The explored NO-delivery reactivity of DNICs include (a) O_2_-induced release of free NO from dinuclear **DNIC–COOH**/**DNIC–COOMe**, (b) O_2_-induced release of free NO from mononuclear DNICs [(NO)_2_Fe(SR)(S_Cys_)]^*n*−^, (c) direct transfer of NO from dinuclear **DNIC–COOH**/**DNIC–COOMe** to deoxyMb, (d) direct transfer of NO^–^ from mononuclear DNICs [(NO)_2_Fe(SR)(S_Cys_)]^*n*−^ to metMb, and (e) direct transfer of NO from mononuclear DNICs [(NO)_2_Fe(SR)(S_Cys_)]^*n*−^ to deoxyMb. In an attempt to elucidate the cellular uptake and intracellular transformations of DNICs as well as intracellular delivery of NO, human cord blood mesenchymal stem cells (MSC), human corneal endothelial cells (HCEC), and neuro-2a cells (N2a) were adopted for further kinetic and mechanistic investigations.

### Kinetic, Mechanistic, and Efficacy Investigations on Cellular Uptake of Dinuclear DNICs, Intracellular Transformation of DNICs, Delivery of NO, and Activation of HO-1

Inspired by the reported binding of dinuclear DNIC [(NO)_2_Fe(μ-SCH_2_CH_2_OH)_2_Fe(NO)_2_] toward BSA yielding an albumin-bound DNIC,^50^ reactions between dinuclear **DNIC–COOH**/**DNIC–COOMe** with BSA under alternative cell culturing media (or PBS at pH 7.4) were investigated before *in vitro* cell experiments. Chemical compositions of alternative cell culturing media and fetal bovine serum (FBS) are collected in Tables S3–S4. α-minimum essential medium (αMEM) with 20% FBS is used for culturing MSC, minimum essential medium (MEM) with 5% FBS is used for culturing N2a, and human endothelial serum-free medium (HSFM) with 2% FBS is used for culturing HCEC. In comparison with the EPR-silent nature of **DNIC–COOH**/**DNIC–COOMe** in cell culturing media, addition of (a) 2% FBS to HFSM, (b) 5% FBS to MEM, or (c) 20% FBS to αMEM resulted in the formation of distinctive EPR signals at *g*_av_ = 2.03 (Figures S3–S4 and Tables 2 and S2). Similar to the interactions between DNICs and Cys-containing proteins reported in the literature, these formations of distinctive EPR signals supported the assembly of albumin-bound DNIC [(NO)_2_Fe(SR)(S_Cys-albumin_)]^*n*−^ (Schemes 1f and 2a). As opposed to **DNIC–COOH**, **DNIC–COOMe** exhibits a rapid binding to BSA, yielding albumin-bound DNIC (Figure S5a–c). Through direct comparisons of the apparent intensity of these EPR features (Figure S3), moreover, elevated formation of albumin-bound DNIC along with the increased %FBS demonstrated a reversible interaction between dinuclear **DNIC–COOH**/**DNIC–COOMe** and BSA leading to the generation of albumin-bound DNIC. As shown in Figure S4, pretreatment of *N*-ethylmaleimide (NEM, a thiol-blocking reagent) to FBS followed by addition of dinuclear **DNIC–COOH**/**DNIC–COOMe** into cell culturing media with NEM-treated FBS resulted in the disappearance of distinctive EPR signals.^50,72^ That is, the Cys-34 (the only thiol in BSA) may serve as the anchoring site for the assembly of albumin-bound DNIC similar to that derived from the reaction of dinuclear DNIC [(NO)_2_Fe(μ-SCH_2_CH_2_OH)_2_Fe(NO)_2_] and BSA.^50^ On the other hand, assembly of albumin-bound DNIC [(NO)_2_Fe(SCH_2_CH_2_COOH)(S_Cys-albumin_)]^−^ via the reaction between 50 mg/mL of BSA and **DNIC–COOH** was also evidenced by the shift of IR ν_NO_ absorption peaks from (1781, 1756) cm^–1^ to (1777, 1747) cm^–1^ (Figure S3g). Based on the investigations discussed above, the hydrophobic nature of **DNIC–COOMe** (Log *D*7.4 = 1.0), in contrast to the hydrophilic nature of deprotonated **DNIC–COOH** at a neutral pH environment (Log *D*7.4 = −2.3), may explain its strong and rapid binding to Cys-34 in the hydrophobic pocket of BSA.^73,74^ Considering that the p*K*_a_ for the carboxylic acid group of HSCH_2_CH_2_COOH is 4.3,^75^ of importance, (partial) formation of intact **DNIC–COOH** at lower pH conditions (i.e., pH = 6.5–4.5 in the endosome/lysosome and pH = 6.8–6.2 under ischemic tissue) may recover its lipophilic nature (Log *P* = 1.2).^76−78^

Regarding the assembly of albumin-bound DNIC derived from the reaction of dinuclear **DNIC–COOH**/**DNIC–COOMe** with BSA, potential NO-delivery reactivity of albumin-bound DNIC was further explored. As shown in Figure S5d–f, delayed decays of the EPR signals at *g*_av_ = 2.03 were observed after incubation of dinuclear **DNIC–COOH**/**DNIC–COOMe** in cell culturing media with FBS under hypoxia conditions (∼1% O_2_). In contrast, the accelerated degradations of albumin-bound DNICs in cell culturing media with FBS under normoxia conditions suggested an O_2_-induced mechanism for the release of NO (Scheme 1g and Figure S5g). During the reaction of metMb with dinuclear **DNIC–COOH**/**DNIC–COOMe** in the presence of BSA, no change of UV–vis absorption bands at (409, 504, 631) nm excluded the NO^–^-transfer reaction despite the formation of mononuclear DNIC [(NO)_2_Fe(SR)(S_Cys-albumin_)]^*n*−^ (Figure S6). As opposed to the NO^–^-transfer reactivity of mononuclear DNIC [(NO)_2_Fe(SR)(S_Cys_)]^*n*−^ to metMb, this absent NO^–^-transfer reactivity of albumin-bound DNIC [(NO)_2_Fe(SR)(S_Cys-albumin_)]^*n*−^ may be ascribed to the steric hindrance between metMb and albumin.

Based on the cell viability study shown in Figure S7, both **DNIC–COOH** and **DNIC–COOMe** displayed a dose-dependent cell-proliferation effect on N2a/HCEC, whereas **DNIC–COOH** features an improved biocompatibility with MSC/N2a/HCEC in comparison with **DNIC–COOMe**. Accordingly, treatment of 100 μM of **DNIC–COOH** (or 2.5 μM of **DNIC–COOMe**) to MSC, treatment of 100 μM of **DNIC–COOH** (or 10 μM of **DNIC–COOMe**) to N2a, and treatment of 75 μM of **DNIC–COOH** (or 1 μM of **DNIC–COOMe**) to HCEC were determined as the optimal conditions for further *in vitro* investigations.

**Figure 4:**
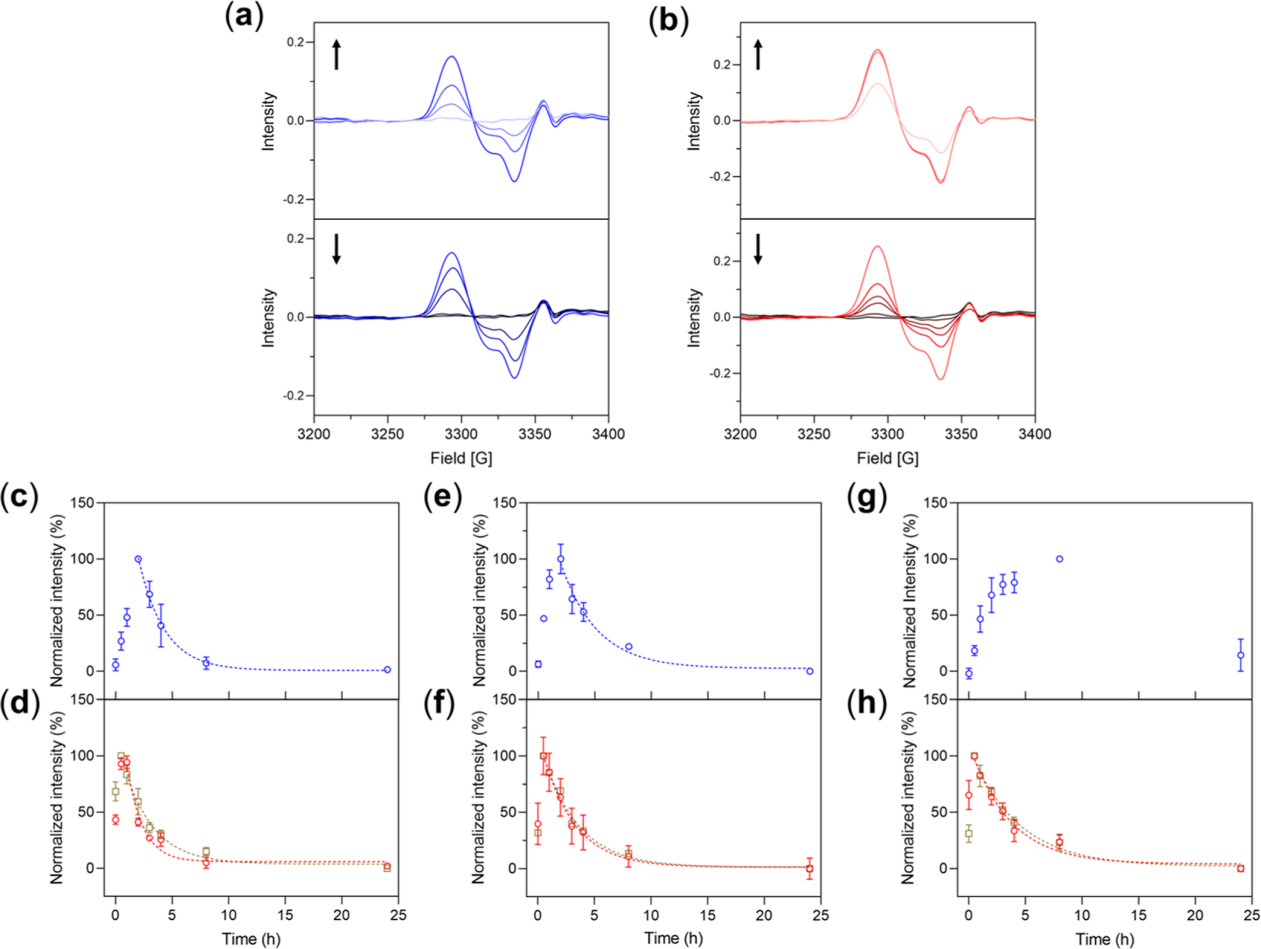
Time-dependent change of EPR spectra for MSCs treated with (a) **DNIC–COOH** (blue) and (b) **DNIC–COOMe** (red). Formation and decay of intracellular mononuclear DNIC upon treatment of **DNIC–COOH** (blue) and **DNIC–COOMe** (red) to (c, d) MSCs, (e, f) N2a, and (g, h) HCEC, respectively, whereas the decay is fitted to pseudo-first-order kinetics (dashed line). Data show the mean ± standard error of the mean (SEM) (*n* = 3). EPR monitoring on mononuclear DNIC derived from the treatments of **DNIC–COOMe** under hypoxia conditions is depicted in gold in the corresponding figures.

Upon treatment of **DNIC–COOH** to MSC, formation of distinctive EPR signal at *g* = 2.041, 2.034, and 2.015 observed in the whole-cell EPR spectra indicated the cellular uptake of **DNIC–COOH** followed by intracellular transformation into mononuclear DNIC [(NO)_2_Fe(SR)(SR′)]^n–^ (Figure 4a and Tables 2 and S2). Based on the reactivity study discussed above and other reported literature, intracellular Cys or other Cys-containing proteins are proposed to facilitate this assembly of mononuclear DNIC [(NO)_2_Fe(SCH_2_CH_2_COOH)(SR′)]^*n*−^ (SR′ = S_Cys_ or S_Cys-protein_, Scheme 2b). As shown in Figures 4a,b and S8 and Table 2, effective cellular uptake of dinuclear **DNIC–COOH**/**DNIC–COOMe** by MSC, N2a, and HCEC, respectively, followed by intracellular conversion into mononuclear DNIC [(NO)_2_Fe(SR)(SR′)]^−^ (SR = SCH_2_CH_2_COOH or SCH_2_CH_2_COOMe; SR′ = S_Cys_ or S_Cys-protein_) was evidenced by the generations of similar EPR features at *g*_av_ = 2.03. According to time-dependent change of whole-cell EPR spectra, **DNIC–COOMe** displayed rapid cellular uptake and intracellular transformation processes with *t*_max_ = 0.5 h upon treatments to MSC, N2a, and HCEC, respectively (Figures 4c–h and S8). In contrast, **DNIC–COOH** exhibited delayed kinetics for these processes based on the *t*_max_ = 2 h in MSC/N2a and *t*_max_ = 8 h in HCEC. Moreover, in comparison with the amount of dinuclear DNICs administered to these cells, spin quantitation of the integrated EPR signal intensity at *t*_max_, using mononuclear DNIC [PPN][(NO)_2_Fe(S_5_)] as a standard, determined the formation of ∼1% and ∼20–40% of mononuclear DNICs derived from dinuclear **DNIC–COOH** and **DNIC–COOMe**, respectively, in MSC/N2a/HCEC. Consequently, these distinctive kinetics and efficacy tailored by types of cells and bridging thiolate ligands in dinuclear DNICs further prompted the study aiming to explore mechanisms for cellular uptake of **DNIC–COOMe**/**DNIC–COOH**.

Regarding the explored binding affinity of dinuclear **DNIC–COOMe**/**DNIC–COOH** to Cys and protein-derived Cys residue(s), a thiol-mediated pathway is one of the potential mechanisms for cellular uptake of DNICs. Due to the discovery of efficient cellular uptake of substrates conjugated to thiol-reactive groups, recently, a thiol-mediated pathway involving dynamic covalent interactions between the thiol-reactive groups and exofacial thiols on cell surfaces followed by direct translocation of the conjugated substrates across the plasma membrane was developed.^79−81^ Consequently, NEM was adopted as an inhibitor for the thiol-mediated pathway through pretreatment of NEM to the investigated cells.^72,79−81^ Although the potential cellular uptake of **DNIC–COOMe** via a passive diffusion process, such a pathway is excluded for **DNIC–COOH**, considering its dianionic nature under the cell culturing media with a neutral pH environment. On the other hand, endocytosis of **DNIC–COOH** was examined using reported inhibitors such as chlorpromazine (CPZ), methyl-β-cyclodextrin (MβCD), and genistein.^82−84^ Before the whole-cell EPR study, a cell viability study of MSC/N2a/HCEC against different inhibitors was performed to determine the optimal conditions (Figure S9).

**Figure 5:**
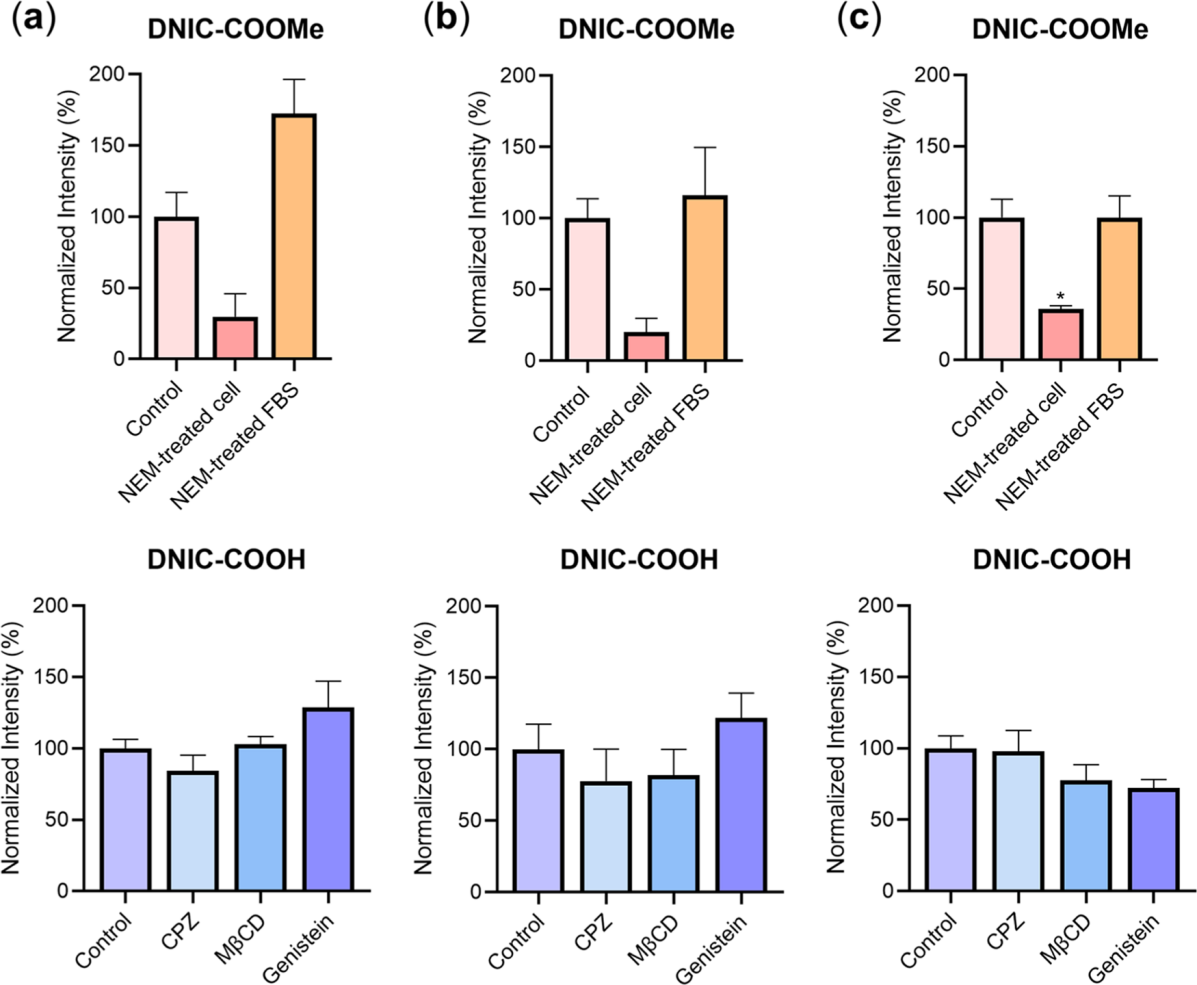
Relative EPR intensity at *g*_av_ = 2.03 for (a) MSC, (b) N2a, and (c) HCEC received sequential treatments of alternative inhibitors and **DNIC–COOMe**/**DNIC–COOH**. The cells that received treatments of only **DNIC–COOMe**/**DNIC–COOH** (control group) are normalized as 100%. Data show the mean ± SEM (*n* = 3). *p < 0.05 for comparison with the control group.

As shown in Figure 5, pretreatment of NEM resulted in a reduction of the whole-cell EPR signal intensity after treatments of **DNIC–COOMe** to MSC (29.7 ± 16.3%, *p* = 0.07), N2a (20.1 ± 9.7%, *p* = 0.07), and HCEC (35.8 ± 2.3%, *p* = 0.01), respectively, for 0.5 h. These results demonstrated a dominant contribution of thiol-mediated mechanism to the cellular uptake of **DNIC–COOMe** (Scheme 2c). Upon treatments of **DNIC–COOMe** to the cells without pretreatments of NEM, of interest, replacement of native FBS with NEM-treated FBS led to increase of the whole-cell EPR signal intensity in MSC (172.4 ± 24.1%, %NEM-treated FBS = 20%) and N2a (116.2 ± 33.6%, %NEM-treated FBS = 5%), while no change is observed in HCEC (99.9 ± 15.3%, %NEM-treated FBS = 2%, Figure 5). As discussed above, treatment of thiol-blocking NEM to FBS inhibited the nucleophilic reactivity of albumin-derived Cys residue and retarded the assembly of albumin-bound DNIC. Upon utilization of NEM-treated FBS, retarded assembly of albumin-bound DNIC in the extracellular environment along with the increase of whole-cell EPR signal intensity excluded albumin-mediated cellular uptake of **DNIC–COOMe**, namely, direct uptake of albumin-bound DNIC was excluded.

During the investigations on **DNIC–COOH** under a similar methodology, no change of whole-cell EPR signal intensity upon pretreatment of NEM and a slight increase of whole-cell EPR signal intensity using NEM-treated FBS excluded the cellular uptake of **DNIC–COOH** through the thiol-/albumin-mediated pathway (Figure S10). Upon sequential treatments of endocytosis inhibitors and **DNIC–COOH** to MSC/N2a/HCEC, of importance, a decrease of whole-cell EPR signal intensity (Figure 5, 84.2 ± 11.0% for CPZ-treated MSC, 77.6 ± 22.5% for CPZ-treated N2a, 81.7 ± 18.2% for MβCD-treated N2a, 77.5 ± 11.1% for MβCD-treated HCEC, 72.1 ± 5.9% for genistein-treated HCEC) may suggest endocytosis as a potential mechanism for cellular uptake of **DNIC–COOH** (Scheme 2d). As opposed to the dominant thiol-mediated cellular uptake of **DNIC–COOMe**, we are aware of the continued efforts required to dissect the comprehensive mechanisms for cellular uptake of **DNIC–COOH** using other synthetic DNICs in combination with alternative types of inhibitors. According to the elucidations on thiol-binding reactivity and cellular uptake of dinuclear **DNIC–COOMe**/**DNIC–COOH**, presumably, the types of bridging thiolate ligands in dinuclear DNICs [(NO)_2_Fe(μ-SR)_2_Fe(NO)_2_] may play a critical role to regulate their thiol-binding rate/affinity and to control the kinetics/efficacy/mechanisms for their cellular uptake.

After thiol-mediated uptake of **DNIC–COOMe** (or endocytosis of **DNIC–COOH**) and intracellular assembly of mononuclear DNICs [(NO)_2_Fe(SR)(SR′)]^−^ (SR = SCH_2_CH_2_COOH or SCH_2_CH_2_COOMe; SR′ = S_Cys_ or S_Cys-protein_), intracellular transformation of DNICs and release/transfer of NO were further elucidated. After the maximum intensity for whole-cell EPR signals was reached, the decrease of the whole-cell EPR signals was fitted to a pseudo-first-order kinetic model. Based on the kinetic fitting, the intracellular half-life for mononuclear DNICs [(NO)_2_Fe(SR)(SR′)]^−^ was (a) 1.0 ± 0.2 h in **DNIC–COOMe**-treated MSC, (b) 2.0 ± 0.5 h in **DNIC–COOMe**-treated N2a, (c) 2.5 ± 0.9 h in **DNIC–COOMe**-treated HCEC, (d) 1.7 ± 0.4 h in **DNIC–COOH**-treated MSC, and (e) 2.1 ± 0.5 h in **DNIC–COOH**-treated N2a (Figure 4c–h, Table 1). As discussed above, (i) O_2_-induced decompositions of mononuclear DNIC [(NO)_2_Fe(SR)(S_Cys_)]^*n*−^ (or [(NO)_2_Fe(SR)(S_Cys-protein_)]^*n*−^) accompanied by release of free NO (Scheme 2e,f) and (ii) direct transfer of NO from mononuclear DNIC [(NO)_2_Fe(SR)(S_Cys_)]^−^ to Fe^2+^-porphyrin protein(s) (i.e., soluble guanylate cyclase (sGC), Scheme 2g) are potential mechanisms for the decrease of whole-cell EPR signal intensity. Consequently, a fluorescence (microscopic) study in combination with DAF-FM (a fluorescence probe for free NO,) as well as an EPR study under hypoxia condition, was further executed.

**Figure 6:**
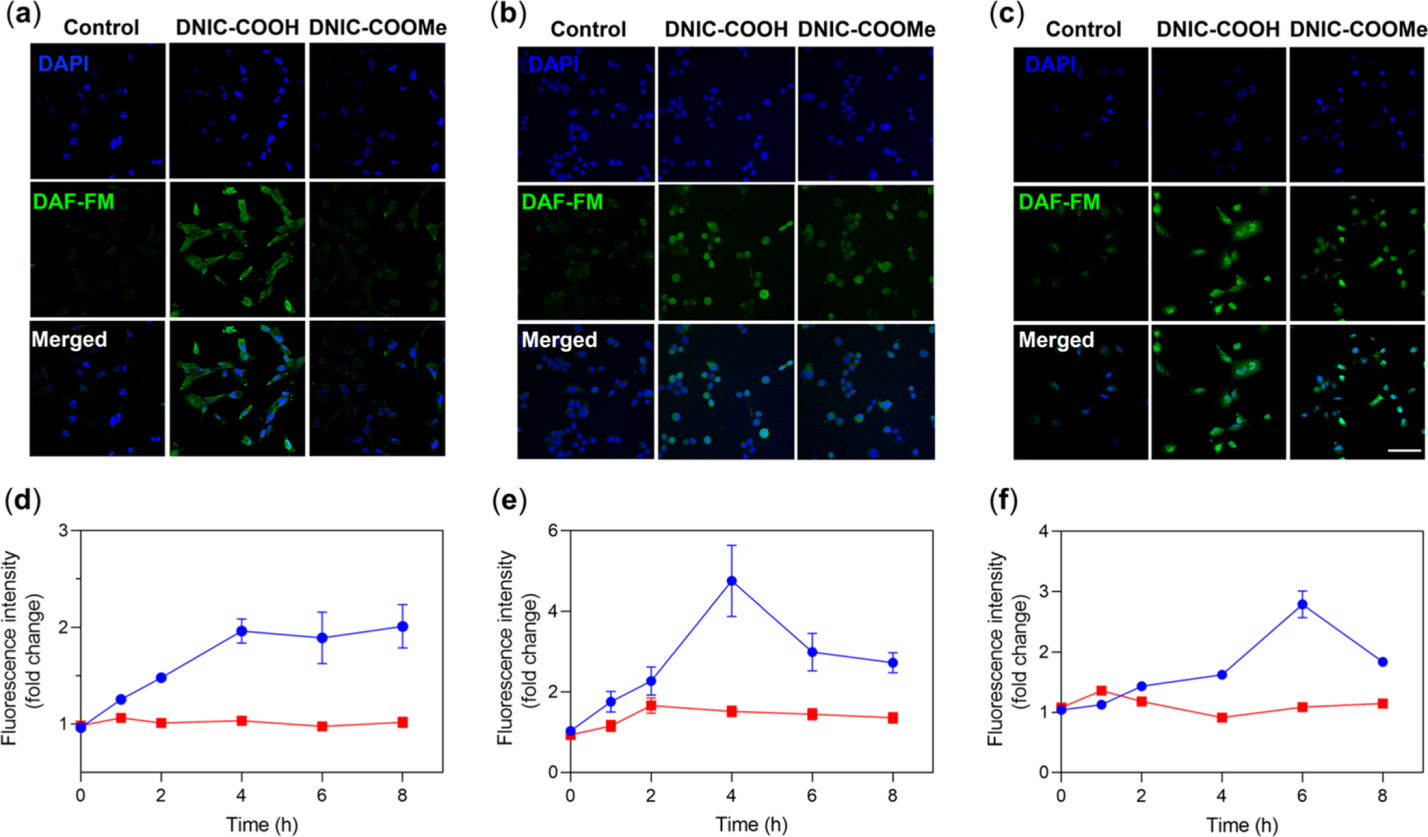
Confocal microscopy images of DAF-FM-treated (a) MSC, (b) N2a, and (c) HCEC without (control group) or with the additional treatments of **DNIC–COOH** and **DNIC–COOMe**, respectively. Scale bar = 50 μm. Kinetics for intracellular release of NO in (d) MSC, (e) N2a, and (f) HCEC treated with **DNIC–COOH** (blue) and **DNIC–COOMe** (red). Data show the mean ± SEM (*n* = 3).

In comparison with the cells treated with only DAF-FM, additional treatments of **DNIC–COOH** (or **DNIC–COOMe**) induced significant formation of fluorescence in the cytoplasm (Figure 6a–c), which indicated the intracellular release of NO. Through monitoring the time-dependent change of intracellular fluorescence intensity, of importance, **DNIC–COOMe** featured a *t*_max_ = 1 h/2 h/1 h for intracellular release of NO in MSC/N2a/HCEC (Figure 6d–f). In comparison, the *t*_max_ for the intracellular release of NO observed in MSC, N2a, and HCEC treated with **DNIC–COOH** were 4, 4, and 6 h, respectively. In combination with the kinetic study using EPR spectroscopy discussed above, these kinetic investigations supported the sequential cellular uptake of dinuclear **DNIC–COOH**/**DNIC–COOMe** via endocytosis/thiol-mediated pathway, intracellular transformation into mononuclear DNIC [(NO)_2_Fe(SR)(S_Cys_)]^*n*−^ (or [(NO)_2_Fe(SR)(S_Cys-protein_)]^*n*−^), and intracellular release of NO (Scheme 2b–f).

After treatment of **DNIC–COOMe** to MSC/N2a/HCEC under hypoxia condition, formation and decomposition of mononuclear DNIC [(NO)_2_Fe(SR)(S_Cys_)]^*n*−^/[(NO)_2_Fe(SR)(S_Cys-protein_)]^*n*−^ were also monitored using EPR spectroscopy. As shown in Figure 4c–h, besides the similar *t*_max_ = 0.5 h for intracellular assembly of mononuclear DNICs, intracellular decompositions of mononuclear DNICs followed *t*_1/2_ = 1.8 ± 0.1 h in MSC, *t*_1/2_ = 2.1 ± 0.4 h in N2a, and *t*_1/2_ = 2.9 ± 0.2 h in HCEC (Table 1). As discussed above, both mononuclear DNICs [(NO)_2_Fe(SR)(S_Cys_)]^*n*−^ and [(NO)_2_Fe(SR)(S_Cys-albumin_)]^*n*−^ displayed an enhanced stability under anaerobic and hypoxia conditions. Consequently, the comparable kinetics for intracellular decompositions of mononuclear DNICs in MSC/N2a/HCEC under normoxia and hypoxia conditions implicated an O_2_-independent process for transfer of NO from mononuclear DNICs to Fe^2+^-porphyrin protein(s) (Scheme 2g). In the attempt to explore this NO-transfer process, confocal microscopic study on activation of cGMP as well as real-time quantitative polymerase chain reaction (qPCR)/enzyme-linked immunosorbent assay (ELISA) analyses on transcriptional/translational regulations of *HMOX1* genes/heme oxygenase (HO)-1 proteins in MSC/N2a/HCEC treated with **DNIC–COOMe**/**DNIC–COOH** were further performed.

**Figure 7:**
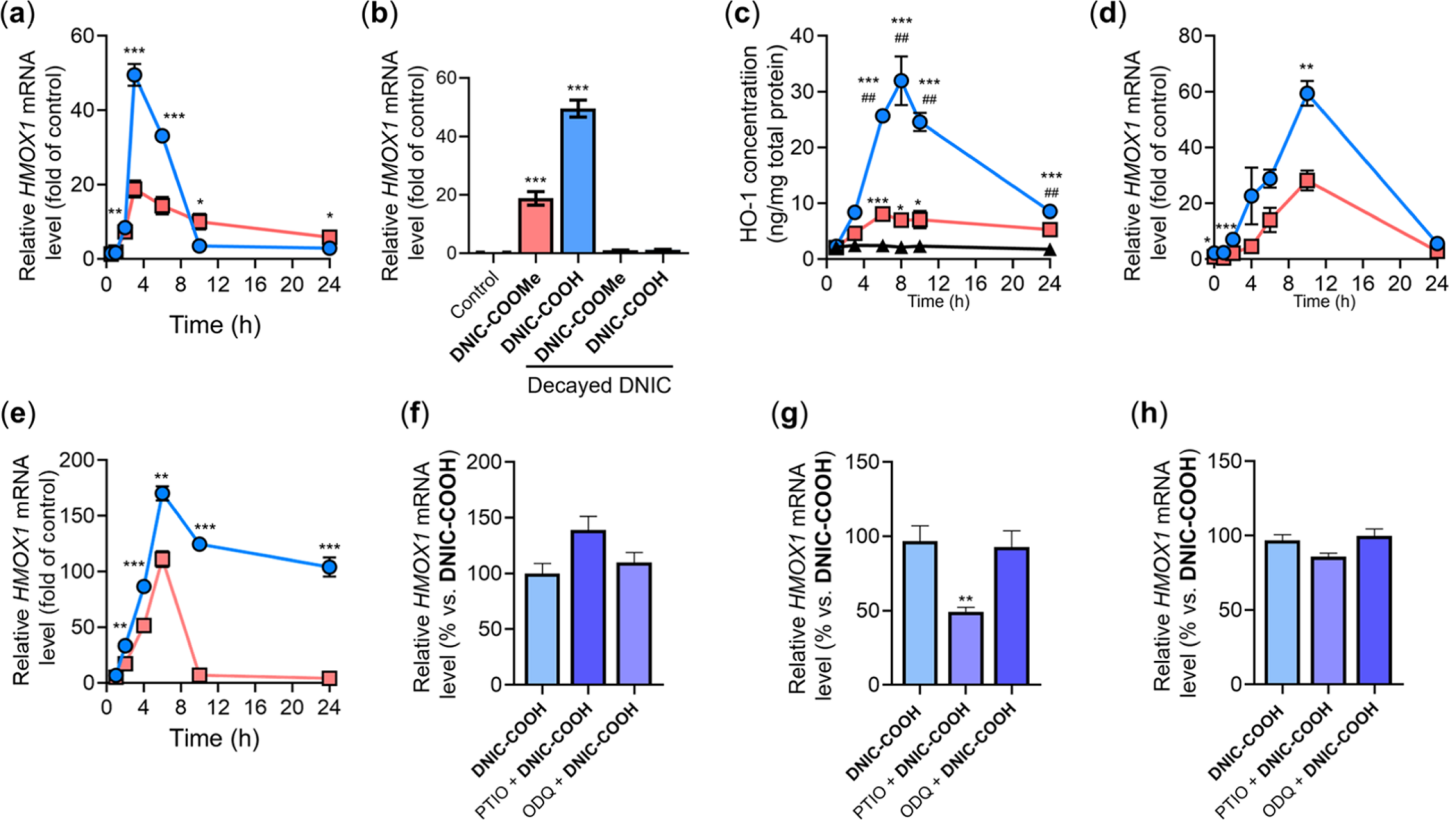
(a) Results of real-time PCR showing the temporal profile of *HMOX1* gene after treatments of **DNIC–COOH** (blue) and **DNIC–COOMe** (red) to MSC. *p < 0.05, **p < 0.01, and ***p < 0.001. (b) *HMOX1* mRNA level in MSC after alternative treatments for 3 h. ***p < 0.001 for comparison with the control group. (c) Results of ELISA showing the temporal profile of intracellular HO-1 protein level without (black) or with treatments of **DNIC–COOH** (blue) and **DNIC–COOMe** (red). *p < 0.05 and ***p < 0.001 for comparison with the control group; ^##^*p* < 0.01 for comparison with the **DNIC–COOMe** group. Results of real-time PCR showing the temporal profile of *HMOX1* gene after treatments of **DNIC–COOH** (blue) and **DNIC–COOMe** (red) to (d) N2a and (e) HCEC. Relative *HMOX1* mRNA level in (f) MSC at 4 h, (g) N2a at 10 h, and (h) HCEC at 6 h after treatments of **DNIC–COOH** without or with the PTIO/ODQ. Data show the mean ± SEM (*n* = 3).

Downstream to the well-documented NO-sGC-cGMP pathway (Scheme 2h,i), HO-1 is a cytoprotective protein of which an elevated expression was reported to enhance the survival of MSC or protection of liver grafts/lung against the oxidative stress under myocardial/diabetic ischemia, ischemic stroke, and after liver/small bowel transplantation.^20−33,36,85,86^ As shown in Figure S11, elevated formation of cGMP observed in the **DNIC–COOMe**-treated (or **DNIC–COOH**-treated) MSC suggested that the DNIC-induced nitrosylation of Fe-porphyrin center in sGC enhanced the conversion of GTP into cGMP. Moreover, treatment of **DNIC–COOH** to MSC triggered maximal 49.5 ± 2.9-fold activation of *HMOX1* gene at 3 h and 32.0 ± 4.4-fold expression of HO-1 protein at 8 h (Figure 7a–c). In comparison, reduced activations of *HMOX1* gene (18.8 ± 2.3-fold at 3 h) and HO-1 protein (8.1 ± 0.9-fold at 6 h) were observed in the **DNIC–COOMe**-treated MSC, although similar kinetic profiles for regulations of *HMOX1* gene and HO-1 protein were observed. Moreover, absent regulations of *HMOX1* gene were observed in the MSC treated with degraded **DNIC–COOH**/**DNIC–COOMe**. Presumably, rapid cellular uptake of **DNIC–COOMe** followed by fast intracellular release/transfer of NO may overwhelm the capacity and kinetics of NO-sGC-cGMP-HO-1 pathway. In contrast, compatible kinetics for intracellular NO-delivery reactivity of **DNIC–COOH** may rationalize the enhanced activations of HO-1. Parallel qPCR investigations on transcriptional regulations of *HMOX1* gene by **DNIC–COOH**/**DNIC–COOMe** in N2a and HCEC were collected in Figure 7d,e. Of importance, **DNIC–COOH**, as opposed to **DNIC–COOMe**, triggered enhanced transcriptional activations of *HMOX1* gene in both N2a and HCEC. In HCEC, of interest, extended activation of *HMOX1* gene up to 24 h may be ascribed to the delayed endocytic uptake of **DNIC–COOH** and steady intracellular delivery of NO (Figure 7e).

Inspired by the effective activation of *HMOX1* gene in **DNIC–COOH**-treated MSC/N2a/HCEC, 2-phenyl-4,4,5,5-tetramethylimidazoline-1-oxyl-3-oxide (PTIO, the scavenger for free NO, Scheme 2j) and 1*H*-[1,2,4]oxadiazolo-[4,3-*a*]quinoxalin-1-one (ODQ, oxidizing reagent for conversion of Fe^2+^-sGC into Fe^3+^-sGC, Scheme 2k) were utilized in the attempt to gain further mechanistic insights.^87−89^ As shown in Figure 7f–h, cotreatment of PTIO to MSC/HCEC and pretreatment of ODQ to MSC/N2a/HCEC, respectively, resulted in no significant perturbation on the transcriptional activation of *HMOX1* gene by **DNIC–COOH**. Regarding the successful scavenging of free NO released from **DNIC–COOH** by PTIO (Figure S12), the null effect of PTIO on **DNIC–COOH**-induced regulation of *HMOX1* gene in MSC/HCEC suggested the importance of NO-transfer reactivity of mononuclear DNIC [(NO)_2_Fe(SR)(S_Cys_)]^*n*−^ (Scheme 2g). That is, direct transfer of NO from mononuclear DNIC [(NO)_2_Fe(SR)(S_Cys_)]^*n*−^ toward Fe^2+^-porphyrin center, instead of the released NO, may trigger activation of sGC for regulation of downstream HO-1. During the treatment of higher concentration of PTIO (100 μM) to N2a, reduced transcriptional activation of *HMOX1* gene by **DNIC–COOH** supports the fact that free NO released from mononuclear DNIC [(NO)_2_Fe(SR)(SR′)]^*n*−^ participates in the modulation of HO-1 in N2a. On the other hand, upon oxidation of the Fe^2+^-porphyrin center within sGC to an Fe^3+^ state by pretreatment with ODQ, retained activation of *HMOX1* gene by **DNIC–COOH** (Figure 7f–h) highlighted the critical role of intracellular assembly of mononuclear DNIC [(NO)_2_Fe(SR)(S_Cys_)]^*n*−^ on direct transfer of NO^–^ to the Fe^3+^-porphyrin center of sGC (Scheme 2g,k). Based on the mechanistic study described above, (a) release of NO from DNICs followed by nitrosylation of Fe^2+^-porphyrin center of sGC and (b) direct transfer of NO/NO^–^ from DNICs to the Fe^2+^/Fe^3+^-porphyrin center of sGC are possible dissociative and associative mechanisms, respectively, for activation of NO-sGC-cGMP-HO-1 pathway.^36,85,86^ Regarding the activation of HO-1, other mechanisms, including (a) NO-sGC-cGMP-Nrf2-HO-1, (b) NO-Keap1/Nrf2-HO-1, and (c) reactive oxygen species (ROS)-Nrf2-HO-1 were also reported,^36,85,86,90,91^ which reveals the potential electrophilic modification of Keap1-derived Cys residue by DNICs (i.e., *S*-nitrosation or *S*-dinitrosylironization).

### Steady Delivery of Intracellular NO and Elevated Activation of Cytoprotective HO-1 by DNIC–COOH Enhance the Survival of MSC/HCEC under Stressed Conditions

**Figure 8:**
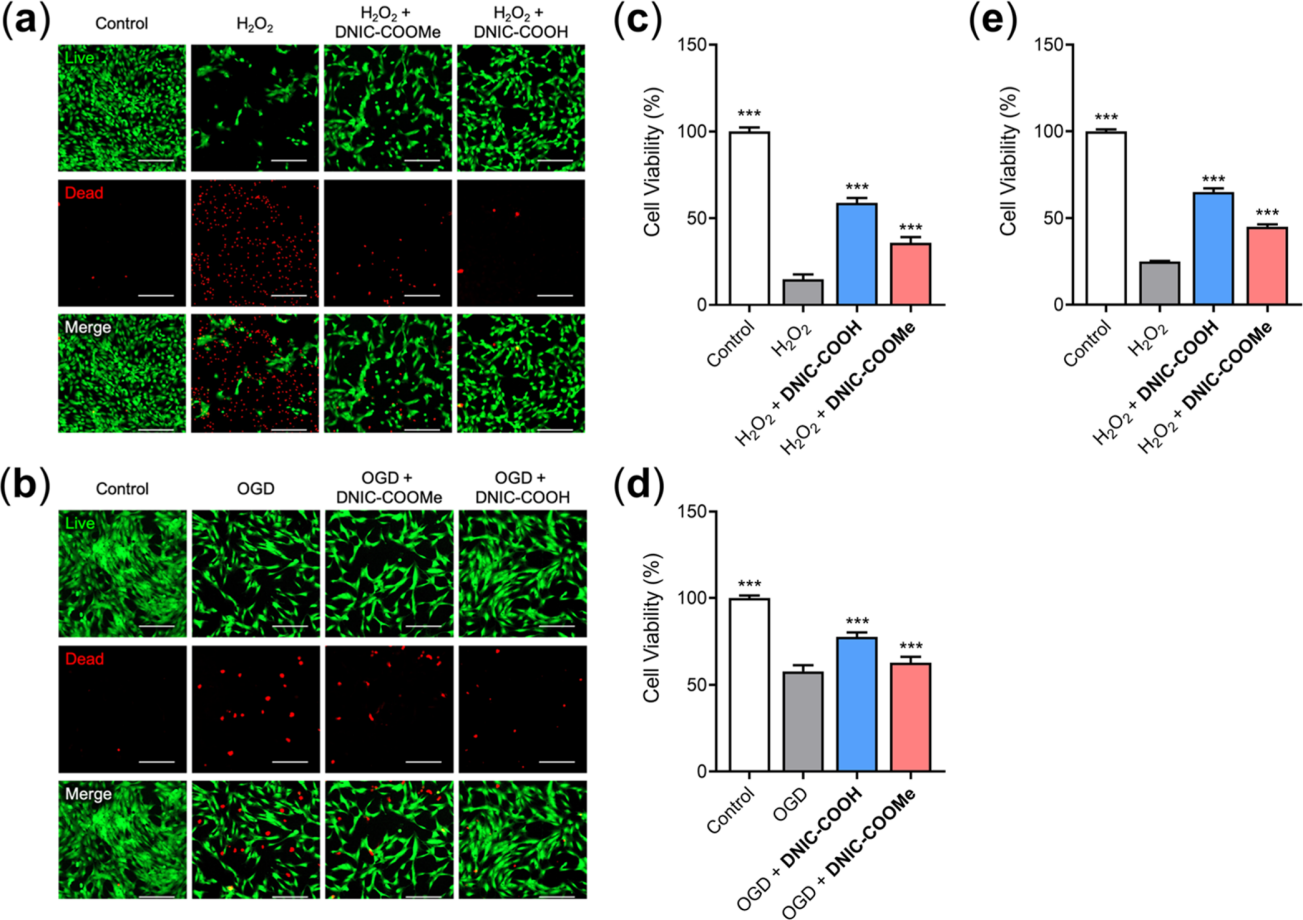
(a, b) Representative live/dead images of MSCs and (c, d) their corresponding cell viability following various treatments. Scale bar, 200 μm. (e) Cell viability of HCEC under alternative treatments. ***p < 0.001 for comparison with the control group.

We then investigated whether the DNIC-induced upregulation of HO-1 was beneficial for the survival of MSCs under stressed conditions. Herein, two *in vitro* models were employed to simulate the hostile microenvironment in the recipient tissues confronted by the MSCs upon transplantation. In the first scenario, hydrogen peroxide (H_2_O_2_) was added to the medium to mimic oxidative stress in inflammatory tissues. MSCs were pretreated with **DNIC–COOMe** or **DNIC–COOH** for 8 h before exposure to H_2_O_2_, while untreated cells and cells exposed to H_2_O_2_ were utilized as controls. As revealed by the results of live/dead staining and CCK-8 assay (Figure 8a,c), significant cell death was observed after H_2_O_2_ treatment, suggesting that the elevated oxidative stress in inflamed tissue may prevent successful engraftment of transplanted cells. In contrast, the viability of MSCs could be significantly improved by pretreatment with DNICs (*p* < 0.005). Furthermore, MSC that received **DNIC–COOH** exhibited a higher survival rate than that of the **DNIC–COOMe** group (58.7% vs 35.8%, *p* < 0.001), indicating the superior cytoprotective potential of **DNIC–COOH** treatment. As shown in Figure 8e, of interest, the **DNIC–COOH**-primed HCEC also displayed an enhanced survival under the mimic oxidative stress condition, which is as opposed to that without or with the pretreatment of **DNIC–COOMe**.

In addition to inflammation-induced oxidative stress, the transplanted cells may also face ischemia-induced hypoxia and limited access to nutrients. To examine the capacity of DNIC in promoting cell survival under such harsh circumstances, cells that were pretreated with **DNIC–COOMe** or **DNIC–COOH** were cultured in an oxygen–glucose-deprived (OGD) condition. Compared with the cells grown under a normal condition, deprivation of oxygen and glucose significantly suppressed cell proliferation and induced cell death (Figure 8b,d), indicating the threat of the ischemic microenvironment to the transplanted cells. The viability of the cells that received **DNIC–COOMe** was comparable to that of OGD group (*p* > 0.05), suggesting that **DNIC–COOMe** preconditioning might not achieve cytoprotective effects on cells under ischemic conditions. For the cells that had been pretreated with **DNIC–COOH**, however, an increase in the number of viable cells was observed, indicating the potential of **DNIC–COOH** in enhancing cell survival in an ischemic microenvironment. Based on the above observations, **DNIC–COOH** exhibited a higher efficiency in upregulating HO-1 expression and a better capacity at improving cell viability under hostile conditions and was therefore employed for the following investigations.

### Conclusions and Comments

In this study, mechanistic, kinetic, and efficacy investigations on the cellular uptake of DNICs, (intracellular) NO-delivery reactivity of DNICs, activation of cytoprotective HO-1, and regulation of enhanced survival of MSC/HCEC under stressed conditions have led to the following results:

1. Based on the IR/EPR/UV–vis spectroscopic investigations and NO-release/NO-transfer reactivity study under normoxia/hypoxia/anaerobic conditions, a variety of NO-delivery mechanisms for dinuclear **DNIC–COOH**/**DNIC–COOMe** were explored. As summarized in Scheme 1, these NO-delivery mechanisms include (a) O_2_-induced release of free NO from dinuclear **DNIC–COOH**/**DNIC–COOMe**, (b) O_2_-induced release of free NO from mononuclear DNIC [(NO)_2_Fe(SR)(S_Cys_)]^*n*−^ or [(NO)_2_Fe(SR)(S_Cys-albumin_)]^*n*−^, (c) direct transfer of NO from dinuclear **DNIC–COOH**/**DNIC–COOMe** to deoxyMb, (d) direct transfer of NO from mononuclear DNIC [(NO)_2_Fe(SR)(S_Cys_)]^*n*−^ to deoxyMb, and (e) direct transfer of NO^–^ from mononuclear DNIC [(NO)_2_Fe(SR)(S_Cys_)]^*n*−^ to metMb.
2. According to the *in vitro* mechanistic/kinetic/efficacy study using EPR spectroscopy in combination with alternative inhibitors, rapid and enhanced binding affinity of **DNIC–COOMe** toward protein-derived Cys residue rationalizes its instantaneous and efficient cellular uptake by MSC/N2a/HCEC (*t*_max_ = 0.5 h) through a thiol-mediated pathway followed by intracellular assembly of mononuclear DNIC [(NO)_2_Fe(SCH_2_CH_2_COOMe)(SR′)]^*n*−^ (SR′ = S_Cys_ or S_Cys-protein_). In contrast, the dianionic nature and retarded Cys-binding capability of **DNIC–COOH** exclude the passive diffusion and thiol-/Cys-/albumin-mediated cellular uptake processes. Using CPZ/MβCD/genistein as endocytic inhibitors, endocytosis is the most plausible mechanism for steady but less efficient cellular uptake of **DNIC–COOH** into MSC/N2a (*t*_max_ = 2 h) and HCEC (*t*_max_ = 8 h).
3. EPR monitoring on the intracellular DNIC [(NO)_2_Fe(SR)(S_Cys_)]^*n*−^/[(NO)_2_Fe(SR)(S_Cys-albumin_)]^*n*−^ unraveled the comparable kinetics for cellular uptake of **DNIC–COOMe** followed by intracellular assembly and decomposition of mononuclear DNICs under normoxia and hypoxia conditions, respectively. The comparable kinetics suggested an O_2_-independent process for associative and direct transfer of NO/NO^–^ from DNICs to Fe^2+^/Fe^3+^-heme protein (i.e., sGC) without transient formation of freely released NO under intracellular environment.
4. Relying on the distinctive mechanisms, kinetics, and efficacy for cellular uptake of dinuclear **DNIC–COOH**/**DNIC–COOMe**, **DNIC–COOMe** features a *t*_max_ of 1–2 h for intracellular release of free NO, while a longer *t*_max_ of 4–6 h for this process was displayed by **DNIC–COOH**. That is, the bridging thiolate ligands in dinuclear **DNIC–COOH**/**DNIC–COOMe** can serve as a switch to modulate the kinetics for O_2_-induced release of NO from intracellular **DNIC–COOH**/**DNIC–COOMe** or DNIC [(NO)_2_Fe(SR)(S_Cys_)]^*n*−^/[(NO)_2_Fe(SR)(S_Cys-albumin_)]^*n*−^.
5. On the basis of qPCR/ELISA analyses and cell viability study, steady endocytic uptake of **DNIC–COOH** and sustained intracellular release/transfer of NO trigger enhanced upregulation of HO-1 and cytoprotective effects. Thus, the survival of **DNIC–COOH**-primed MSC/HCEC was improved under stressed conditions mimicking the hostile microenvironments after cell transplantation.

NO-induced regulation of MSC and activation of HO-1 in other types of cells using NONOates and DNICs were reported in the literature.^92−94^ In comparison with NONOates, synthetic DNICs were explored to be more effective in triggering apoptosis of Jurkat cells and enhancing proliferation/migration/tube formation of EA.hy926 human vascular endothelial cells,^45,95^ which may be ascribed to the different NO-delivery mechanisms. In terms of mechanisms for delivery of NO to the intracellular NO-responsive targets (i.e., Fe-porphyrin center in sGC), a variety of NONOates feature a pH-dependent nature for release of free NO in the extracellular environment through hydrolysis. Subsequent passive diffusion of freely released NO from extracellular environment to intracellular compartment occurs followed by interaction of delivered NO with Fe^2+^-porphyrin center in sGC and regulation of the well-known NO-sGC-cGMP pathway. Despite the wide range of half-life for release of NO from hydrolysis of alternative types of NONOates, the NO-delivery mechanism for NONOates explains (a) the formation of peroxynitrite and cytotoxic ^•^OH/^•^NO_2_ species via a reaction between freely released NO and endogenous O_2_^–^ under pathological condition (i.e., inflammation) and (b) absent activation of NO-sGC-cGMP pathway under oxidative stress condition due to the inert nature of freely released NO toward the Fe^3+^-porphyrin center in sGC.^67,87−89^ In contrast, (a) superoxide-scavenger nature, (b) ^•^OH/^•^NO_2_-free NO-delivery reactivity, (c) modulated mechanism/kinetic/efficacy for cellular uptake, and (d) direct NO/NO^–^-transfer activity (i.e., toward the Fe^2+^/Fe^3+^-porphyrin center of sGC) of dinuclear DNICs [(NO)_2_Fe(μ-SR)_2_Fe(NO)_2_] highlight the potential of synthetic DNICs, as a novel type of NO prodrugs, for development of (MSC-based) regenerative medicine and cell therapy. In addition to DNIC-induced upregulation of cytoprotective HO-1 in MSC explored in this study, potential activation of neurogenesis,^50^ anti-inflammation effect, and blood–brain barrier permeability^44^ by NO-delivery DNICs prompts our undergoing study on the combination of DNICs and MSC for the treatment of ischemic stroke.

## Materials and Methods

### Reagents

The reagents sodium chloride (NaCl) from Merck; potassium chloride (KCl), disodium hydrogen phosphate (Na_2_HPO_4_), and potassium dihydrogen phosphate (KH_2_PO_4_) from Showa; sodium triphosphate (Na_3_PO_4_), deuterium chloride (DCl), *N*-ethylmaleimide (NEM), and 2,4-di*tert*-butyl phenol (DTBP) from ACROS; chlorpromazine (CPZ) from TCI; methyl-β-cyclodextrin (MβCD) from Thermo Scientific; deuterium oxide (D_2_O), met-myoglobin (metMb), l-cysteine (Cys), and 2-phenyl-4,4,5,5-tetramethylimidazoline-1-oxyl-3-oxide (PTIO) from Sigma; genistein from Alfa Aesar; 1*H*-[1,2,4]oxadiazolo-[4,3-*a*]quinoxalin-1-one (ODQ) from Cayman; and 4-amino-5-methylamino-2′,7′-difluorofluorescein diacetate (DAF-FM) from Invitrogen were used. Double-distilled water was obtained using a Millipore water distilling apparatus. Complexes [Fe_2_(μ-SCH_2_CH_2_COOH)_2_(NO)_4_] (**DNIC–COOH**), [Fe_2_(μ-SCH_2_CH_2_COOMe)_2_(NO)_4_] (**DNIC–COOMe**), and [PPN][(NO)_2_Fe(S_5_)] were synthesized based on published procedures.^55,63^

### Instruments

All of the EPR measurements were performed at X-band using a Bruker EMXmicro-6/1/S/L spectrometer equipped with a Bruker E4119001 super high sensitivity cavity. X-band EPR spectra were obtained with a microwave power of 0.6456–0.6348 mW, frequency at 9.41 GHz, conversion time of 66.68 ms, receiver gain of 30, and modulation amplitude of 10.0 G at 100 kHz. UV–vis spectra were recorded on a PerkinElmer Lambda 365 spectrometer. Fourier transform infrared (FT-IR) spectra were recorded using a sealed solution cell (0.1 mm, CaF_2_ windows). Reactions of dinuclear **DNIC–COOH/DNIC–COOMe** with O_2_ in the presence of DTBP were characterized using Trace 1300 Gas Chromatograph in combination with a mass spectrometer with a 5MS column. The confocal microscopic images were recorded using ZEISS LSM 780 or Leica TCS–SP5-X AOBS confocal microscope systems. The absorbance of the assay was recorded using a microplate reader SpectraMax iD3, Molecular Devices, San Jose, CA.

### NO-Release Reactivity of DNICs

The NO-release reactivity of DNICs in 50 mM potassium phosphate buffer (pH 7.4) under normoxia condition at 37 °C was investigated using Nitrate/Nitrite Colorimetric Assay Kit (Item No. 780001, Cayman). After addition of 10 μL of 100 mM stock solution of **DNIC–COOH** (or **DNIC–COOMe**) in DMSO to 990 μL of 50 mM potassium phosphate buffer (pH 7.4), 25 μM of **DNIC–COOH** (or **DNIC–COOMe**) was prepared by the addition of 0.125 mL of this 1 mM solution of **DNIC–COOH** (or **DNIC–COOMe**) to 4.875 mL of 50 mM potassium phosphate buffer (pH 7.4). This solution was incubated at 37 °C under normoxia condition for 0, 1, 2, 4, 8, 24, 48, and 72 h, respectively. Then, 40 μL of the solution was collected to assess the release of NO using a Nitrate/Nitrite Colorimetric Assay Kit (Item No. 780001, Cayman). After each 40 μL aliquot of the aqueous solution was mixed with 40 μL of kit assay buffer, 10 μL of Enzyme Cofactor Mixture (Item No. 780012) and 10 μL of nitrate reductase mixture (Item No. 780010) were added before this mixture solution was incubated at room temperature for 1 h. Subsequently, addition of 50 μL of Griess Reagent R1 (Item No. 780018) and 50 μL of Griess Reagent R2 (Item No. 780020) followed by incubation at room temperature for 15 min results in the formation of UV–vis absorption band at 540 nm. The absorbance at 540 nm was then recorded using a microplate reader (SpectraMax iD3, Molecular Devices, San Jose, CA) with a reference wavelength of 800 nm. According to a calibration curve made with 0, 5, 10, 15, 20, 25, 30, and 35 μM of nitrite standard (Item No. 780016)/nitrate standard (Item No. 780014), respectively, NO-release reactivity of **DNIC–COOH** (or **DNIC–COOMe**) in PBS (pH 7.4) at each time point was further estimated. Assuming that the aerobic degradation of **DNIC–COOH** (or **DNIC–COOMe**) follows pseudo-first order kinetics, the half-life for release of NO from **DNIC–COOH** (or **DNIC–COOMe**) in 50 mM potassium phosphate buffer (pH 7.4) at 37 °C was determined. Three independent experiments were conducted to measure the average half-life for the release of NO from **DNIC–COOH** (or **DNIC–COOMe**).

Under normoxia condition, the NO-release reactivity of 25 μM **DNIC–COOH** (or **DNIC–COOMe**) (a) with the presence of 200 μM of Cys in 50 mM potassium phosphate buffer (pH 7.4), (b) in α-minimum essential medium (αMEM, Thermo Fisher Scientific, Waltham, MA) without/with the presence of 20% fetal bovine serum (FBS, Gibco), (c) in minimum essential medium (MEM, Hyclone) without/with the presence of 5% FBS, and (d) in human endothelial serum-free medium (HSFM, Gibco) without/with the presence of 2% FBS was evaluated using Nitrate/Nitrite Colorimetric Assay Kit (Item No. 780001, Cayman) under a similar procedure described above.

### Reaction of DNIC–COOH (or DNIC–COOMe) with O_2(g)_ in the Presence of 2,4-Di-*tert*-butyl Phenol (DTBP)

To a 25 mL Schlenk tube loaded with 5 mL of THF mixture solution of **DNIC–COOH** (4.4 mg, 0.01 mmol) and DTBP (8.3 mg, 0.04 mmol), 2.4 mL of O_2(g)_ (0.1 mmol) was added via a gastight syringe at ambient temperature. After this THF mixture solution was stirred overnight, it was used for gas chromatography–mass spectrometry (GC–MS) analysis. The reaction of **DNIC–COOMe** and 10 equiv of O_2(g)_ in the presence of 4 equiv of DTBP followed by GC-MS analysis was performed under a similar procedure.

### Reactions of DNICs with Cys in PBS (pH 7.4) under Normoxia or Anaerobic Condition

50 μM of **DNIC–COOH** was prepared via addition of 1 μL of 100 mM stock solution of **DNIC–COOH** in DMSO to 2 mL of PBS (pH 7.4) with the presence of 10 mM Cys. After this solution was incubated at 37 °C for 0, 1, 2, and 4 h, respectively, EPR spectra of 100 μL aliquots of this solution were measured. Moreover, double integration of the EPR signal was executed to obtain the integrated EPR signal intensity under each condition, which was further utilized to characterize the time-dependent formation and degradation of Cys-bound mononuclear DNIC [(NO)_2_Fe(SCH_2_CH_2_COOH)(S_Cys_)]^*n*−^. EPR investigations on the formation and degradation of the Cys-bound DNICs upon incubation of 50 μM of **DNIC–COOMe** in PBS (pH 7.4) with the presence of 10 mM of Cys were performed in a similar manner.

Reactions of **DNIC–COOH**/**DNIC–COOMe** with Cys under anaerobic condition were performed under a similar procedure, while all of the samples were prepared under an anaerobic N_2(g)_ atmosphere, and the PBS buffer (pH 7.4) was degassed before use.

### Reactions of DNICs with metMb/deoxyMb without or with the Presence of Cys (or BSA)

After addition of 30 μL of 500 μM stock solution of **DNIC–COOH** (in DMSO) to a 4 mL quartz cuvette containing 5 μM metMb and 200 μM Cys in 3 mL of 25 mM PBS (pH 7.4), UV–vis spectra for this mixture solution were measured every 30 min. Transformation of deoxyMb into MbNO was achieved after reaction for 4 h based on the complete shift of UV–vis absorption bands from (410, 502, and 629) nm to (419, 548, and 578) nm.

Reactions of (a) 5 μM of **DNIC–COOMe** with 5 μM of metMb in the presence of 200 μM of Cys, (b) 5 μM of **DNIC–COOH**/**DNIC–COOMe** with 5 μM of deoxyMb in the presence of 200 μM of Cys, and (c) 5 μM of **DNIC–COOH**/**DNIC–COOMe** with 5 μM of metMb in the presence of 50 mg/mL BSA were performed under a similar manner.

### EPR Investigations on Reactions of DNICs under Alternative Cell Culturing Media without/with the Presence of FBS

#### Under Normoxia Condition

50 μM of **DNIC–COOH** was prepared via addition of 1 μL of 100 mM stock solution of **DNIC–COOH** in DMSO to 2 mL of αMEM with the presence of 20% FBS. After this solution was incubated at 37 °C for 0, 0.5, 1, 2, 3, 4, 8, and 24 h, respectively, EPR spectra of 100 μL aliquots of this solution were measured. Moreover, double integration of the EPR signal was executed to obtain the integrated EPR signal intensity under each condition, which was further utilized to characterize the time-dependent formation and degradation of BSA-bound DNIC. EPR investigations on the formation and degradation of protein-bound DNICs upon incubation of (1) 50 μM **DNIC–COOMe** in αMEM with the presence of 20% FBS, (2) 50 μM **DNIC–COOH** (or **DNIC–COOMe**) in MEM with the presence of 5% FBS, and (3) 50 μM **DNIC–COOH** (or **DNIC–COOMe**) in HSFM with the presence of 2% FBS were performed in a similar manner. In addition, EPR spectra for 50 μM of **DNIC–COOH** (or 50 μM of **DNIC–COOMe**) in αMEM, MEM, and HSFM, respectively, were also measured.

#### Thiol-Blocking Experiments Using *N*-Ethylmaleimide (NEM)

After a 35 mg/mL solution of NEM was formulated in pure FBS and incubated at 4 °C overnight, the NEM-treated FBS (NEM-FBS) was used to prepare αMEM supplemented with 20% NEM-FBS. Then, 1 μL of 100 mM stock solution of **DNIC–COOH** in DMSO was added to 2 mL of αMEM with the presence of 20% NEM-FBS (50 μM of **DNIC–COOH**) and incubated at 37 °C for 4 h before the measurement of its respective EPR spectrum. EPR spectra for (1) 50 μM **DNIC–COOH** in MEM with the presence of 5% NEM-FBS, (2) 50 μM **DNIC–COOH** in HSFM with the presence of 2% NEM-FBS, (3) 50 μM **DNIC–COOMe** in αMEM with the presence of 20% NEM-FBS, (4) 50 μM **DNIC–COOMe** in MEM with the presence of 5% NEM-FBS, and (5) 50 μM **DNIC–COOMe** in HSFM with the presence of 2% NEM-FBS were measured following a similar procedure.

#### Under Hypoxia Condition

EPR investigations on the formation and degradation of albumin-bound DNICs upon incubation under hypoxia condition were performed following the procedure described below. αMEM with the presence of 20% FBS were incubated under a hypoxia condition (1% oxygen and 5% CO_2_ atmosphere in a hypoxia incubator, Heracell VIOS 160i, Thermo Fisher Scientific) overnight. On the next day, 50 μM **DNIC–COOH** was prepared via addition of 1 μL of 100 mM stock solution of **DNIC–COOH** in DMSO to 2 mL of the preconditioned αMEM with the presence of 20% FBS. After incubation of this solution at 37 °C under hypoxia condition for 8 and 24 h, respectively, EPR spectra of 100 μL aliquots were measured. In a similar manner, EPR investigations on (1) 50 μM **DNIC–COOH** in MEM with the presence of 5% FBS, (2) 50 μM **DNIC–COOH** in HSFM with the presence of 2% FBS, (3) 50 μM **DNIC–COOMe** in αMEM with the presence of 20% FBS, (4) 50 μM **DNIC–COOMe** in MEM with the presence of 5% FBS, and (5) 50 μM **DNIC–COOMe** in HSFM with the presence of 2% FBS were performed.

### Reactions of DNICs with BSA in PBS (pH 7.4) Monitored Using FT-IR

5 μL of DMSO stock solution of 100 mM **DNIC–COOH** was transferred to a 20 mL sample vial loaded with 50 mg of BSA in 0.995 mL of 25 mM deuterated sodium phosphate buffer (pH 7.4). This reaction solution was then monitored by FT-IR after incubation under anaerobic condition at ambient temperature for 1 h. The shift of IR νNO stretching peaks from (1781, 1756) cm^–1^ to (1777, 1747) cm^–1^ suggested the formation of albumin-bound DNIC [(NO)_2_Fe(SCH_2_CH_2_COOH)(S_Cys-albumin_)]^−^.

### Determination of Log *D*7.4 for DNICs

PBS (pH 7.4) and *n*-octanol were fully degassed before use. After adding 20 μL of 100 mM stock solution of **DNIC–COOH** in DMSO to 19.98 mL of PBS (pH 7.4), 2 mL of this 100 μM **DNIC–COOH** solution was added with 2 mL of *n*-octanol. This mixture solution was vortexed thoroughly for 30 s and incubated until the two solutions were separated. UV–vis spectra of **DNIC–COOH** in PBS (pH 7.4) and *n*-octanol were measured in order to determine Log *D*7.4 using the equation shown below:^96^
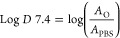
*A*_O_ is the *A*_362_ in *n*-octanol, and *A*_PBS_ is the *A*_362_ in PBS (pH 7.4) after partitioning. Log *D*7.4 for **DNIC–COOMe** was determined in a similar manner. On the other hand, the Log *P* value for **DNIC–COOH** was measured based on its distribution in ddH_2_O and *n*-octanol using a similar procedure.^97,98^

### Cell Culture

Human umbilical cord blood mesenchymal stem cells (MSC) were purchased from the Bioresource Collection and Research Center, Food Industry Research and Development Institute (Hsinchu, Taiwan) and maintained in αMEM (Thermo Fisher Scientific, Waltham, MA USA) supplemented with 20% FBS (Gibco), 30 mg/mL hygromycin B (Thermo Fisher Scientific), and 4 ng/mL basic fibroblast growth factor (bFGF; Thermo Fisher Scientific). Mouse neuroblast cells (N2a) were cultured in MEM (Hyclone) supplemented with 5% FBS, 1% sodium pyruvate (Gibco), and 1% penicillin/streptomycin (Invitrogen). Human corneal endothelial cells (HCEC) were maintained in human endothelial serum-free medium (HSFM; Gibco) supplemented with 2% FBS and 10 ng/mL bFGF. The cells were cultured at 37 °C with 5% CO_2_ in a humidified incubator.

### Cell Viability

Cell viability study of MSC, N2a, and HCEC cells with or without the treatment of **DNIC–COOH** was performed using a Cell Counting Kit-8 (IMT Formosa New Materials Co., Ltd., Taiwan) according to the manufacturer’s protocol. Briefly, cells were seeded into a 96-well plate at a density of 4 × 10^3^ cells/well for MSC, 1 × 10^4^ cells/well for N2a, and 8 × 10^3^ cells/well for HCEC, respectively, and incubated overnight. On the next day, cells were treated with **DNIC–COOH** at different concentrations and incubated for 24 h. The culture medium was then removed before the cells were washed with DPBS. After addition of fresh medium containing CCK-8 solution to the cells and incubation for 1–3 h, the absorbance at 450 nm was then measured on a microplate reader (SpectraMax iD3, Molecular Devices, San Jose, CA) with a reference wavelength of 650 nm. Three independent experiments were executed, and the results were represented as mean ± SEM% (*n* = 3) with the untreated cells (control) as 100% viability. Cell viability studies of MSC/N2a/HCEC cells (1) with the treatment of **DNIC–COOMe** for 24 h, (2) with the treatment of NEM for 0.5 h, (3) with the treatment of chlorpromazine (CPZ; TCI) for 0.5 h, (4) with the treatment of methyl-β-cyclodextrin (MβCD; Thermo Fisher Scientific) for 0.5 h, (5) with the treatment of genistein (Alfa Chemistry) for 0.5 h, (6) with the sequential treatments of ODQ for 1 h and **DNIC–COOH** for 3 h (MSC)/10 h (N2a)/6 h (HCEC), and (7) with the cotreatment of PTIO and **DNIC–COOH** for 3 h (MSC)/10 h (N2a)/6 h (HCEC), respectively, were performed in a similar manner. In addition to CCK-8 assay, a live/dead staining was performed using a ViaQuant Viability/Cytotoxicity Kit (GeneCopoeia, Rockville, MD) according to the manufacturer’s instruction.

### EPR Investigations on Cellular Uptake of DNICs and Intracellular Formation/Decomposition of Protein-Bound DNICs in MSC/N2a/HCEC

#### Under Normoxia Condition

Cellular uptake of **DNIC–COOH** and intracellular formation/decomposition of mononuclear DNICs in MSC/N2a/HCEC cells were investigated using EPR spectroscopy. Briefly, cells were seeded into two (or one) 100 mm dish(es) at a density of 2 × 10^6^ cells/dish and incubated at 37 °C overnight. On the next day, cells were treated with 100 μM of **DNIC–COOH** (or 100 μM of **DNIC–COOH** to N2a and 75 μM of **DNIC–COOH** to HCEC) and incubated for 0, 0.5, 1, 2, 3, 4, 8, and 24 h, respectively. After removal of the supernatant solution, cells were washed with DPBS before addition of 1 mL of trypsin (0.05%)/EDTA (0.53 mM) and incubation for 5 min at 37 °C. After the obtained cell suspension solutions from two 100 mm dishes were combined and centrifuged at 100*g* for 5 min, the supernatant was removed before addition of 100 μL of αMEM to DNIC-treated MSC (or MEM to DNIC-treated N2a and HSFM to DNIC-treated HCEC). This solution was then transferred into EPR quartz tube and frozen in N_2(l)_ before the EPR measurements. For each measured EPR spectrum, double integration was executed to obtain the integrated EPR intensity under each condition. Moreover, time-dependent decay of integrated EPR signal intensity after treatment of **DNIC–COOH** was fit to pseudo-first-order kinetics in order to determine the intracellular half-life for EPR-active mononuclear DNIC. Three independent experiments were executed to measure the average half-life for intracellular decomposition of EPR-active mononuclear DNIC after treatment with **DNIC–COOH** at 37 °C. On the other hand, at the time point with maximum integrated EPR signal intensity, spin quantitation of intracellular EPR-active DNICs was performed using mononuclear DNIC [PPN][(NO)_2_Fe(S_5_)] as a standard according to the methodology used in the previous work.^50^ Cellular uptake of **DNIC–COOMe** (2.5 μM to MSC, 10 μM to N2a, or 1 μM to HCEC) and intracellular formation/decomposition of EPR-active mononuclear DNICs were investigated using EPR spectroscopy in a similar manner.

#### Pretreatments of Inhibitors

Studies of cellular uptake mechanisms were investigated using EPR spectroscopy in combination with pretreatments of alternative inhibitors. After the MSC cells were seeded into two (or one) 100 mm dish(es) at a density of 2 × 10^6^ cells/dish and incubated at 37 °C overnight, sequential treatments of 50 μM of NEM for 0.5 h and 100 μM of **DNIC–COOH** for 2 h were performed before the treated MSC cells were collected for EPR measurement following the procedure described above. Under a similar procedure, (1) N2a cells received sequential treatments of 25 μM of NEM for 0.5 h and 100 μM of **DNIC–COOH** for 2 h, (2) HCEC cells received sequential treatments of 50 μM of NEM for 0.5 h and 75 μM of **DNIC–COOH** for 6 h, (3) MSC cells received sequential treatments of 50 μM of NEM for 0.5 h and 2.5 μM of **DNIC–COOMe** for 0.5 h, (4) N2a cells received sequential treatments of 50 μM of NEM for 0.5 h and 10 μM of **DNIC–COOMe** for 0.5 h, (5) HCEC cells received sequential treatments of 50 μM of NEM for 0.5 h and 1 μM of **DNIC–COOMe** for 0.5 h, (6) MSC cells received sequential treatments of 10 μM of CPZ for 0.5 h and 100 μM of **DNIC–COOH** for 0.5 h, (7) N2a cells received sequential treatments of 10 μM of CPZ for 0.5 h and 100 μM of **DNIC–COOH** for 0.5 h, (8) HCEC cells received sequential treatments of 10 μM of CPZ for 0.5 h and 75 μM of **DNIC–COOH** for 0.5 h, (9) MSC cells received sequential treatments of 10 mM of MβCD for 0.5 h and 100 μM of **DNIC–COOH** for 0.5 h, (10) N2a cells received sequential treatments of 10 mM of MβCD for 0.5 h and 100 μM of **DNIC–COOH** for 0.5 h, (11) HCEC cells received sequential treatments of 40 mM of MβCD for 0.5 h and 75 μM of **DNIC–COOH** for 0.5 h, (12) MSC cells received sequential treatments of 200 μM of genestein for 0.5 h and 100 μM of **DNIC–COOH** for 0.5 h, (13) N2a cells received sequential treatments of 200 μM of genestein for 0.5 h and 100 μM of **DNIC–COOH** for 0.5 h, and (14) HCEC cells that received sequential treatments of 200 μM of genestein for 0.5 h and 75 μM of **DNIC–COOH** for 0.5 h were prepared and collected for EPR measurements.

#### Under Hypoxia Condition

Similar to the methodology described above, cellular uptake of **DNIC–COOMe** and intracellular formation/decomposition of mononuclear DNICs in MSC/N2a/HCEC cells under hypoxia condition (1% oxygen and 5% CO_2_ atmosphere in a hypoxia incubator, Heracell VIOS 160i, Thermo Fisher Scientific) were investigated using EPR spectroscopy. Time-dependent decay of integrated EPR signal intensity after treatment of **DNIC–COOMe** was fit to pseudo-first-order kinetics in order to determine the intracellular half-life for EPR-active mononuclear DNIC under hypoxia condition. Three independent experiments were executed to measure the average half-life for intracellular decomposition of EPR-active mononuclear DNIC after treatment with **DNIC–COOMe** at 37 °C under hypoxia condition.

### Kinetic Study on Intracellular Release of NO in MSC/N2a/HCEC Cells after Treatment of DNICs

Intracellular release of NO by **DNIC–COOH** was validated using the fluorescence probe 4-amino-5-methylamino-2′,7′-difluorofluorescein diacetate (DAF-FM) in combination with fluorescence analysis techniques. Briefly, the MSC cells (or N2a and HCEC cells) were seeded onto a 96-well black plate at a density of 6 × 10^3^ cells/well (or 1 × 10^4^ cells/well for N2a and 8 × 10^3^ cells/well for HCEC cells) and incubated at 37 °C overnight. On the next day, the MSC cells were first treated with 10 μM of DAF-FM (or 5 μM for N2a and HCEC cells) and incubated at 37 °C for 0.5 h. After removal of the supernatant solution, 100 μM of **DNIC–COOH** was added to the MSC cells (or 100 μM of **DNIC–COOH** to N2a cells and 75 μM of **DNIC–COOH** to HCEC cells) and incubated for 0, 1, 2, 4, 6, and 8 h, respectively, in the dark. Fluorescence intensity of the cells was then recorded using a microplate reader (SpectraMax iD3, Molecular Devices, San Jose, CA) with an excitation wavelength at 485 nm and an emission wavelength at 525 nm. Three independent experiments were executed to measure the time-dependent change of average fluorescence intensity. Time-dependent change of average fluorescence intensity for the MSC/N2a/HCEC cells with sequential treatment of DAF-FM (10 μM for MSC cells, 5 μM for N2a cells, 5 μM for HCEC cells; 0.5 h) and **DNIC–COOMe** (2.5 μM for MSC cells, 10 μM for N2a cells, 1 μM for HCEC cells) was determined in a similar manner.

Confocal microscopy images for the MSC/N2a/HCEC cells that received sequential treatment of DAF-FM and **DNIC–COOH** (or **DNIC–COOMe** were taken following the procedure described below. Cells were plated in 24-well plates at a density of 2.5 × 10^4^ cells/well for MSC cells, 6 × 10^4^ cells/well for N2a cells, and 2.5 × 10^4^ cells/well for HCEC cells, respectively, on cover glasses (18 mm in diameter) for 24 h and incubated with the DAF-FM (10 μM for MSC cells, 5 μM for N2a cells, 5 μM for HCEC cells) for 0.5 h. After the cell culturing media were removed, **DNIC–COOH** (500 μM for MSC cells, 100 μM for N2a cells, 75 μM for MSC cells) was then added and incubated for 1 h (MSC), 2 h (N2a), and 6 h (HCEC), respectively. Then, the supernatant solution was removed, and the treated cells were washed thrice with DPBS before the cells were fixed with 4% formaldehyde solution for 15 min at room temperature. After the cells were washed thrice with DPBS followed by membrane permeabilization with 0.25% Triton for 5 min, the cover glasses containing fixed cells were washed thrice with DPBS and mounted onto a microscope slide containing 10 μL of mounting medium (FluoroQuest) with 4′-6-diamidino-2-phenylindole (DAPI) for cell nuclei staining. The optical images were captured using a confocal imaging system (ZEISS, LSM 780). Confocal microscopy images of the MSC/N2a/HCEC cells with sequential treatment of DAF-FM (10 μM and 0.5 h for MSC, 5 μM and 0.5 h for N2a, 5 μM and 0.5 h for HCEC) and **DNIC–COOMe** (12.5 μM and 1 h for MSC, 10 μM and 0.5 h for N2a, 1 μM and 0.5 h for HCEC) were taken under a similar procedure.

### Investigation of DNIC-Induced cGMP Formation

The MSC cells were plated on a cover glass (18 mm in diameter) loaded into a 24-well plate at a density of 1.7 × 10^3^ cells/well until the desired confluency was reached. After the culture media were removed, 100 μM of **DNIC–COOH** (or 2.5 μM of **DNIC–COOMe**) was added to the cell culture and incubated for 2 h. The culture media were removed before the MSC cells were washed three times with PBS, whereas the washed MSC cells were further fixed with 4% paraformaldehyde (PFA) solution for 15 min at room temperature. After the PFA solution was removed, the MSC cells were washed twice with PBS for 3 min and permeabilized using 0.1% Triton X-100 (in PBS) at room temperature for 4 min. Subsequently, the MSC cells were washed twice with PBS for 3 min and incubated with a blocking buffer (2.5% BSA in PBS) at room temperature for 30 min. Then, the MSC cells were incubated with primary antibody solution (1:1000 for rabbit anti-cGMP antibody, purchased from Biorbyt, Cambridge, U.K.) at room temperature for 1 h before the MSC cells were washed three times with PBS for 3 min. Cover glasses containing the fixed MSC cells were mounted onto a microscope slide loaded with Fluoroshield mounting medium with DAPI to stain the nuclei before the optical images were captured using a confocal imaging system (Leica TCS-SP-X AOBS).

### Investigation of DNIC-Induced HO-1 Expression

After treatment of (1) 100 μM of **DNIC–COOH** to MSC, (2) 100 μM of **DNIC–COOH** to N2a, (3) 75 μM of **DNIC–COOH** to HCEC, (4) 2.5 μM of **DNIC–COOMe** to MSC, (5) 10 μM of **DNIC–COOMe** to N2a, and (6) 1 μM of **DNIC–COOMe** to HCEC, respectively, the total RNA of the treated cells was extracted using TRIzol Reagent (Thermo Fisher Scientific) in order to assess the time-dependent change in the mRNA level of HO-1. After the synthesis of complementary DNA with the High-Capacity Reverse Transcription Kit, qPCR was conducted using Power SYBR Green PCR Master Mix with the StepOnePlus Real-Time PCR System (Thermo Fisher Scientific). The primer sequences were as follows: *HMOX1* forward 5′-AACTTTCAGAAGGGCCAGGT-3′ and reverse 5′-CTGGGCTCTCCTTGTTGC-3′; *RPL13A* forward 5′-CATAGGAAGCTGGGAGCAAG-3′ and reverse 5′- GCCCTCCAATCAGTCTTCTG-3′; *GAPDH* forward 5′-AATCCCATCACCATCTTCCA-3′ and reverse 5′-TGGACTCCACGACGTACTCA-3′. The relative mRNA levels of target genes were determined by a comparative threshold cycle method and normalized to that of *RPL13A* (or GAPDH). The mRNA level of HO-1 in the MSC, N2a, and HCEC cells, respectively, that received the (1) sequential treatments of 1*H*-[1,2,4]oxadiazolo-[4,3-*a*]quinoxalin-1-one (ODQ, 10 μM for 1 h) and **DNIC–COOH** (100 μM in MSC for 3 h, 100 μM in N2a for 10 h, and 75 μM in HCEC for 6 h) or (2) cotreatment of 2-phenyl-4,4,5,5-tetramethylimidazoline-1-oxyl-3-oxide (PTIO, 100 μM in MSC, 200 μM in N2a, and 100 μM in HCEC) and **DNIC–COOH** (100 μM in MSC for 3 h, 100 μM in N2a for 10 h, and 75 μM in HCEC for 6 h) was also evaluated under a similar procedure.

After the treatments of **DNIC–COOH**/**DNIC–COOMe** to the MSC cells following the procedure described above, the intracellular level of HO-1 protein in DNIC-treated MSC cells was also determined by ELISA (Abcam, Cambridge, MA) according to the manufacturers’ protocols.

### Assessment of DNIC-Induced Cytoprotective Effect in MSCs and HCECs against Hostile Microenvironment

To investigate the capacity of DNIC preconditioning to protect the MSC cells against elevated oxidative stress, cells were pretreated with 2.5 μM of **DNIC–COOMe** or 100 μM of **DNIC–COOH** for 8 h before being incubated with 250 μM H_2_O_2_ for another 24 h. Cell viability was evaluated by live/dead staining or CCK-8 assay. In a similar manner, DNIC-induced cytoprotective effect in HCECs was evaluated through pretreatment of 1 μM of **DNIC–COOMe** or 75 μM of **DNIC–COOH** for 24 h.

To simulate the microenvironment of ischemic tissues, the MSC cells with or without DNIC priming were cultured using glucose-free DMEM in a humidified hypoxia incubator (1% oxygen; Heracell VIOS 160i, Thermo Fisher Scientific) for 48 h. Cell viability was assessed by live/dead staining or CCK-8 assay.

### Statistical Analysis

Data are expressed as the mean ± SEM for all of the *in vitro* study and the mean ± SD for the rest. Statistical analyses were performed using GraphPad Prism software (version 8.2; San Diego, CA). For a comparison of two groups, an unpaired, two-tailed Student’s *t* test was used. One-way analysis of variance (ANOVA) with Tukey’s correction was employed for comparisons of three or more groups. Differences were considered to be significant at *p* < 0.05.

## Supporting Information Available

Additional experimental results, including UV–vis, EPR, IR, NO-release reactivity, cell viability assay, and chemical compositions of minimum essential media (MEM), minimum essential medium α (αMEM), and fetal bovine serum (FBS) (PDF)
