# Supplementary material for: Mechanistic and Kinetic Insights into Cellular Uptake of Biomimetic Dinitrosyl Iron Complexes and Intracellular Delivery of NO for Activation of Cytoprotective HO-1
Source: JACS Au. 2024 Mar 29;4(4):1550–69. doi: 10.1021/jacsau.4c00064 (PMC11040670; doi:10.1021/jacsau.4c00064)
Supplement: Supplementary file 1 — au4c00064_si_001.pdf [file au4c00064_si_001.pdf]

Supporting Information

Mechanistic and Kinetic Insights into Cellular Uptake of Biomimetic Dinitrosyl Iron Complexes and Intracellular Delivery of NO for Activation of Cytoprotective HO-1

Han Chiu,[1,8] Anyelina Chau Fang,[1,8] Yi-Hong Chen,[1,8] Ru Xin Koi,[1] Kai-Ching Yu,[1] Li-Hung Hsieh,[1] Yueh-Ming Shyu,[1] Tarik Abdelkareem Mostafa Amer,[2] Yi-Jen Hsueh,[3] Yu-Ting Tsao,[3] Yang-Jin Shen,[4,5] Yun-Ming Wang,[2] Hung-Chi Chen,[3,4*] Yu-Jen Lu,[4,5*] Chieh-Cheng Huang,[1*] Tsai-Te Lu[1,6,7*]

Affiliations:

[1]Institute of Biomedical Engineering, National Tsing Hua University, Hsinchu, 30013 Taiwan.

[2]Department of Biological Science and Technology, Institute of Molecular Medicine and Bioengineering, College of Biological Science and Technology, National Yang Ming Chiao Tung University, Hsinchu 300, Taiwan.

[3]Department of Ophthalmology and Center for Tissue Engineering, Chang Gung Memorial Hospital, Taoyuan 33305, Taiwan.

[4]College of Medicine, Chang Gung University, Kwei-San, Taoyuan, 33302, Taiwan.

[5]Department of Neurosurgery, Chang Gung Memorial Hospital, Taoyuan 33305, Taiwan.

[6]Department of Chemistry, National Tsing Hua University, Hsinchu, 30013 Taiwan.

[7]Department of Chemistry, Chung Yuan Christian University, Taoyuan 32023, Taiwan.

[8]These authors contributed equally: Ms. Han Chiu, Ms. Anyelina Chau Fang, and Mr. Yi-Hong Chen.

[*]**Corresponding author:**

Hung-Chi Chen, M.D. Ph.D., Department of Ophthalmology and Center for Tissue Engineering, Chang Gung Memorial Hospital, Taoyuan, Taiwan; Department of Medicine, College of Medicine, Chang Gung University, Taoyuan, Taiwan. E-mail: mr3756@cgmh.org.tw.

Yu-Jen Lu, M.D. Ph.D., Department of Neurosurgery, Chang Gung Memorial Hospital, College of Medicine Chang Gung University, Taoyuan, 33305, Taiwan; College of Medicine, Chang Gung University, Kwei-San, Taoyuan, 33302, Taiwan. E-mail: alexlu0416@gmail.com.

Chieh-Cheng Huang, Ph.D., Institute of Biomedical Engineering, National Tsing Hua University, Hsinchu, 30013 Taiwan. E-mail: chiehcheng@mx.nthu.edu.tw.

Tsai-Te Lu, Ph.D., Institute of Biomedical Engineering and Department of Chemistry, National Tsing Hua University, Hsinchu, 30013 Taiwan. E-mail: ttlu@mx.nthu.edu.tw

Table S1. Kinetics for NO-delivery Reactivity and Degradation of DNICs under Alternative Conditions.

NO-delivery Reactivity of DNICs					
DNIC Precursors	Generated DNICs	Media	Additives	Conditions	$t_{1/2}$ or Reaction Time (h)
**DNIC-COOH**	**DNIC-COOH**[a]	Phosphate Buffer (pH 7.4)	-	Normoxia	10.7 ± 1.4[e]
	$[(NO)_2Fe(SR)(S_{Cys})]^{n-}$ [b]	Phosphate Buffer (pH 7.4)	Cys[a]	Normoxia	1.2 ± 0.1[e]
	**DNIC-COOH**	Phosphate Buffer (pH 7.4)	deoxyMb[a]	Anaerobic	-
	$[(NO)_2Fe(SR)(S_{Cys})]^{n-}$ [b]	Phosphate Buffer (pH 7.4)	deoxyMb/Cys[a]	Anaerobic	3.5[f]
	$[(NO)_2Fe(SR)(S_{Cys})]^{n-}$ [b]	Phosphate Buffer (pH 7.4)	metMb/Cys[a]	Anaerobic	4.0[f]
**DNIC-COOMe**	**DNIC-COOMe**[a]	Phosphate Buffer (pH 7.4)	-	Normoxia	9.1 ± 0.6[e]
	$[(NO)_2Fe(SR)(S_{Cys})]^{n-}$ [b]	Phosphate Buffer (pH 7.4)	Cys[a]	Normoxia	1.2 ± 0.2[e]
	**DNIC-COOMe**	Phosphate Buffer (pH 7.4)	deoxyMb[a]	Anaerobic	-
	$[(NO)_2Fe(SR)(S_{Cys})]^{n-}$ [b]	Phosphate Buffer (pH 7.4)	deoxyMb/Cys[a]	Anaerobic	2.0[f]
	$[(NO)_2Fe(SR)(S_{Cys})]^{n-}$ [b]	Phosphate Buffer (pH 7.4)	metMb/Cys[a]	Anaerobic	4.0[f]
Degradation of DNICs					
DNIC Precursors	Generated DNICs	Media	Additives	Conditions	$t_{1/2}$ (h) or rate constant[g]
**DNIC-COOH**	$[(NO)_2Fe(SR)(S_{Cys})]^{n-}$ [b]	Phosphate Buffer (pH 7.4)	Cys[a]	Normoxia	0.7 ± 0.1
	$[(NO)_2Fe(SR)(S_{Cys\text{-}albumin})]^{n-}$ [c]	αMEM	20% FBS	Normoxia	4.8 ± 0.7
		MEM	5% FBS	Normoxia	2.8 ± 0.5
		HSFM	2% FBS	Normoxia	6.5 ± 2.0
	$[(NO)_2Fe(SR)(S_{Cys})]^{n-}$ or $[(NO)_2Fe(SR)(S_{Cys\text{-}protein})]^{n-}$ [d]	MSC	-	Normoxia	1.7 ± 0.4
		N2a	-	Normoxia	2.1 ± 0.5
**DNIC-COOMe**	$[(NO)_2Fe(SR)(S_{Cys})]^{n-}$ [b]	Phosphate Buffer (pH 7.4)	Cys[a]	Normoxia	0.5 ± 0.1
	$[(NO)_2Fe(SR)(S_{Cys\text{-}albumin})]^{n-}$ [c]	αMEM	20% FBS	Normoxia	3.5 ± 0.5
		MEM	5% FBS	Normoxia	1.8 ± 0.7
		HSFM	2% FBS	Normoxia	1.5 ± 0.5
	$[(NO)_2Fe(SR)(S_{Cys})]^{n-}$ or $[(NO)_2Fe(SR)(S_{Cys\text{-}protein})]^{n-}$ [d]	MSC	-	Normoxia	1.0 ± 0.2
				Hypoxia	1.8 ± 0.1
		N2a	-	Normoxia	2.0 ± 0.5
				Hypoxia	2.1 ± 0.4
		HCEC	-	Normoxia	2.5 ± 0.9
				Hypoxia	2.9 ± 0.2

$[(NO)_2Fe(\mu\text{-}SC_6H_3Cl_2)_2(NO)_2]$	$[(NO)_2Fe(SR)(S_{GS})]^{n-}$	Tris-HCl buffer (pH 7.0)	GSH	Normoxia	$k = 5.6\times10^{-5}$ s^{-1} [i,1]
	$[(NO)_2Fe(SR)(S_{Cys\text{-}albumin})]^{n-}$	Tris-HCl buffer (pH 7.0)	albumin	Normoxia	$k = 3.9\times10^{-4}$ and 2.3×10^{-5} s^{-1} [j,1]
	$[(NO)_2Fe(SR)(S_{Cys\text{-}mucin})]^{n-}$	Tris-HCl buffer (pH 7.0)	mucin	Normoxia	$k = 2.6\times10^{-5}$ s^{-1} [i,1]
$[Fe(SC(NH_2)_2)_2(NO)_2]_2$ $[Fe_2(S_2O_3)_2(NO)_4]$	$[(NO)_2Fe(S_{Cys\text{-}albumin})(N_{His\text{-}albumin})]^{n-}$	Tris-HCl buffer (pH 7.0)	albumin	Normoxia	$k = 3.9\times10^{-4}$ and 2.3×10^{-5} s^{-1} [j,2]
$[Fe_2(S_2O_3)_2(NO)_4]^{2-}$	$[(NO)_2Fe(S_{Cys\text{-}albumin})(N_{His\text{-}albumin})]^{n-}$	Tris-HCl buffer (pH 7.0)	albumin	Anaerobic	$k = 4.0\times10^{-6}$ s^{-1} [i,3]
DNDGIC[h]	$[(NO)_2Fe(S_{GS})(S_{Cys\text{-}Prx1})]^{n-}$	Phosphate Buffer (pH 7.4)	Prx 1	Normoxia	$k = 7.0\pm0.4$ $M^{-1}s^{-1}$ [k,4]
	-	Rat Liver	-	Normoxia	4.5[5]
	-	Human Placenta	-	Normoxia	8.0[5]

[a]Concentrations of the reactants are: **DNIC-COOH** = 25 μM, **DNIC-COOMe** = 25 μM, deoxyMb = 5 μM, and metMb = 5 μM. 200 μM of Cys was used for study of NO-delivery reactivity of DNICs, while 10 mM of Cys was used for study of degradation of DNICs. [b]Obtained from reaction of **DNIC-COOH**/**DNIC-COOMe** and Cys. [c]Obtained from reaction of **DNIC-COOH**/**DNIC-COOMe** and BSA in αMEM with 20% FBS, MEM with 5% FBS, or HSFM with 2% FBS. [d]Obtained from treatments of **DNIC-COOH**/**DNIC-COOMe** to MSC, N2a, or HCEC. [e]Half-life ($t_{1/2}$) for release of NO from DNICs monitored using total nitrate/nitrite assay. [f]Reaction time for complete conversion of deoxyMb/metMb into MbNO monitored using UV-vis spectroscopy. [g]Half-life ($t_{1/2}$) for degradation of DNICs monitored using EPR or UV-vis spectroscopy. [h]DNDGIC = $[(NO)_2Fe(S_{GS})_2]^{n-}$ and $[(NO)_2Fe(\mu\text{-}S_{GS})_2Fe(NO)_2]^{m-}$ (GS = deprotonated glutathione). [i]first-order rate constant. [j]first-order rate constants for two parallel reactions. [k]second-order rate constant.

Table S2. EPR Parameters for Cys-/protein-bound Mononuclear DNICs.

DNIC Precursors	Generated DNICs	Additives	Media	EPR Parameters (g_1, g_2, g_3 or g_{av})
**DNIC-COOH**	$[(NO)_2Fe(SR)(S_{Cys})]^{n-}$ [a]	Cys	PBS (pH 7.4)	(2.041, 2.035, 2.015)
	$[(NO)_2Fe(SR)(S_{Cys\text{-}albumin})]^{n-}$ [b]	20% FBS	aMEM	(2.044, 2.037, 2.015)
		5% FBS	MEM	(2.042, 2.036, 2.015)
		2% FBS	HSFM	(2.042, 2.035, 2.014)
	$[(NO)_2Fe(SR)(S_{Cys})]^{n-}$ or $[(NO)_2Fe(SR)(S_{Cys\text{-}protein})]^{n-}$ [c]	-	MSC	(2.041, 2.034, 2.015)
		-	N2a	(2.041, 2.034, 2.014)
		-	HCEC	(2.040, 2.034, 2.015)
**DNIC-COOMe**	$[(NO)_2Fe(SR)(S_{Cys})]^{n-}$ [a]	Cys	PBS (pH 7.4)	(2.041, 2.035, 2.014)
	$[(NO)_2Fe(SR)(S_{Cys\text{-}albumin})]^{n-}$ [b]	20% FBS	aMEM	(2.043, 2.036, 2.015)
		5% FBS	MEM	(2.042, 2.036, 2.015)
		2% FBS	HSFM	(2.042, 2.035, 2.014)
	$[(NO)_2Fe(SR)(S_{Cys})]^{n-}$ or $[(NO)_2Fe(SR)(S_{Cys\text{-}protein})]^{n-}$ [c]	-	MSC	(2.041, 2.035, 2.015)
		-	N2a	(2.041, 2.034, 2.014)
		-	HCEC	(2.040, 2.034, 2.014)
$[(NO)_2Fe(S(CH_2)_2OH)(S(CH_2)_2NH_3)]$	-	-	H_2O	(2.042, 2.029, 2.014)[6]
	$[(NO)_2Fe(SR)(S_{Cys})]^{n-}$ or $[(NO)_2Fe(SR)(S_{Cys\text{-}protein})]^{n-}$	-	SKBR-3	(2.03)[6]
B-DNIC-GSH[d]	$[(NO)_2Fe(SR)(S_{Cys})]^{n-}$ or $[(NO)_2Fe(SR)(S_{Cys\text{-}protein})]^{n-}$	-	Rat Lung	(2.04, 2.03, 2.014)[7]
		-	Abdominal Muscle Tissue	(2.04, 2.01, 2.0) [8]
B-DNIC-NAC[d]	$[(NO)_2Fe(SR)(S_{Cys})]^{n-}$ or $[(NO)_2Fe(SR)(S_{Cys\text{-}protein})]^{n-}$	-	Rat Lung	(2.04, 2.03, 2.014)[7]
$[(NO)_2Fe(S_{Cys})_2]^{n-}$	-	-	HEPES (pH 7.4)	(2.03)[9]
DNDGIC[d]	$[(NO)_2Fe(S_{GS})(S_{Cys\text{-}Prx1})]^{n-}$	Prx 1	Phosphate Buffer (pH 7.4)	(2.040, 2.029, 2.016)[4]
$[(NO)_2Fe(\mu\text{-}SC_6H_3Cl_2)_2(NO)_2]$	$[(NO)_2Fe(SR)(S_{Cys\text{-}albumin})]^{n-}$	albumin	Tris-HCl buffer (pH 7.0)	(2.036, 2.016)[1]
	$[(NO)_2Fe(SR)(S_{Cys\text{-}mucin})]^{n-}$	mucin	Tris-HCl buffer (pH 7.0)	(2.033)[1]

$[(NO)_2Fe(\mu\text{-}SGluc)_2(NO)_2]^d$	$[(NO)_2Fe(S_{Gluc})(S_{GS})]^{n-}$	GSH	phenol-free DMEM-F12	(2.02)[10]
	$[(NO)_2Fe(S_{Gluc})(N_{HisF})]^{n-}$	HisF	phenol-free DMEM-F12	(2.02)[10]

[a]Obtained from reaction of **DNIC-COOH**/**DNIC-COOMe** and Cys. [b]Obtained from reaction of **DNIC-COOH**/**DNIC-COOMe** and BSA in aMEM with 20% FBS, MEM with 5% FBS, or HSFM with 2% FBS. [c]Obtained from treatments of **DNIC-COOH**/**DNIC-COOMe** to MSC, N2a, or HCEC. [d]B-DNIC-GSH = $[(NO)_2Fe(\mu\text{-}S_{GS})_2Fe(NO)_2]^{m-}$ (GS = deprotonated glutathione), B-DNIC-NAC = $[(NO)_2Fe(\mu\text{-}S_{NAC})_2Fe(NO)_2]^{m-}$ (NAC = deprotonated N-acetyl cysteine), DNDGIC = $[(NO)_2Fe(S_{GS})_2]^{n-}$ and $[(NO)_2Fe(\mu\text{-}S_{GS})_2Fe(NO)_2]^{m-}$ (GS = deprotonated glutathione), SGluc = 1-thio-β-D-glucose tetraacetate.

Table S3. Chemical Composition of Minimum Essential Media (MEM) and Minimum Essential Medium Alpha (αMEM).

Component	MEM	αMEM
pH	~7.4	~7.4
Cl^- (mM)	127.6	127.0
HCO_3^-/H_2CO_3 (mM)	26.2	26.2
$HPO_4^{2-}/H_2PO_4^-$(mM)	1.0	1.0
SO_4^{2-} (mM)	0.8	0.8
Na^+ (mM)	144.8	145.9
K^+ (mM)	5.3	5.3
Ca^{2+} (mM)	1.8	1.8
Mg^{2+} (mM)	0.8	0.8
Glucose (mM)	5.6	5.6
Sodium Pyruvate (mM)	-	1
Lipoic Acid (μM)	-	0.97
**Amino acids**		
Glycine (mM)	-	0.67
L-Alanine (mM)	-	0.28
L-Arginine (mM)	0.60	0.50
L-Asparagine (mM)	-	0.33
L-Aspartic acid (mM)	-	0.23
L-Cysteine (mM)	-	0.57
L-Cystine (mM)	0.10	0.10
L-Glutamic Acid (mM)	-	0.51
L-Glutamine (mM)	-	2.00
L-Histidine (mM)	0.20	0.20
L-Isoleucine (mM)	0.40	0.40
L-Leucine (mM)	0.40	0.40
L-Lysine (mM)	0.40	0.40
L-Methionine (mM)	0.10	0.10
L-Phenylalanine (mM)	0.19	0.19
L-Proline (mM)	-	0.35
L-Serine (mM)	-	0.24
L-Threonine (mM)	0.40	0.40
L-Tryptophan (mM)	0.05	0.05
L-Tyrosine (mM)	0.20	0.23
L-Valine (mM)	0.39	0.39
**Vitamins**		
Ascorbic Acid (mM)	-	0.28
Biotin (mM)	-	0.0004
Choline (mM)	0.0071	0.0071
D-Calcium pantothenate (mM)	0.0021	0.0021
Folic Acid (mM)	0.0023	0.0023
Niacinamide (mM)	0.0082	0.0082
Pyridoxal (mM)	0.0049	0.0049
Riboflavin (mM)	0.0003	0.0003
Thiamine (mM)	0.0030	0.0030
Vitamin B12 (mM)	-	0.001
i-Inositol (mM)	0.011	0.011
**Ribonucleosides**		
Adenosine (mM)	-	0.04
Cytidine (mM)	-	0.04

Guanosine (mM)	-	0.04
Uridine (mM)	-	0.04
**Deoxyribonucleosides**		
2'Deoxyadenosine (mM)	-	0.04
2'Deoxycytidine (mM)	-	0.04
2'Deoxyguanosine (mM)	-	0.04
Thymidine (mM)	-	0.04

Table S4. Major Chemical Composition of Fetal Bovine Serum (FBS).

Component	100% FBS	20% FBS	5% FBS	2% FBS
pH	6.5 - 8.5	6.5 - 8.5	6.5 - 8.5	6.5 - 8.5
Cl^- (mM)	99	19.8	4.95	1.98
Phosphorus (mM)	3.07	0.61	0.1535	0.0614
Na^+ (mM)	136	27.2	6.8	2.72
K^+ (mM)	>10	>2	>0.5	>0.2
Ca^{2+} (mM)	3.38	0.68	0.169	0.0676
Iron (μM)	29.7	5.94	1.485	0.594
Glucose (mM)	5.5	1.1	0.275	0.11
Protein (total, mg/mL)	35	7	1.75	0.7
Albumin (mg/mL)	21	4.2	1.05	0.42
Urea (mM)	2.33	0.47	0.1165	0.0466
Bilirubin (mg/L)	3	0.6	0.15	0.06
Cholesterol (mg/L)	300	60	15	6
Creatinine (mg/L)	25	5	1.25	0.5
Globulin (mg/L)	14	2.8	0.7	0.28
Hemoglobin (mg/L)	133	26.6	6.65	2.66
IgG (μg/mL)	89	17.8	4.45	1.78

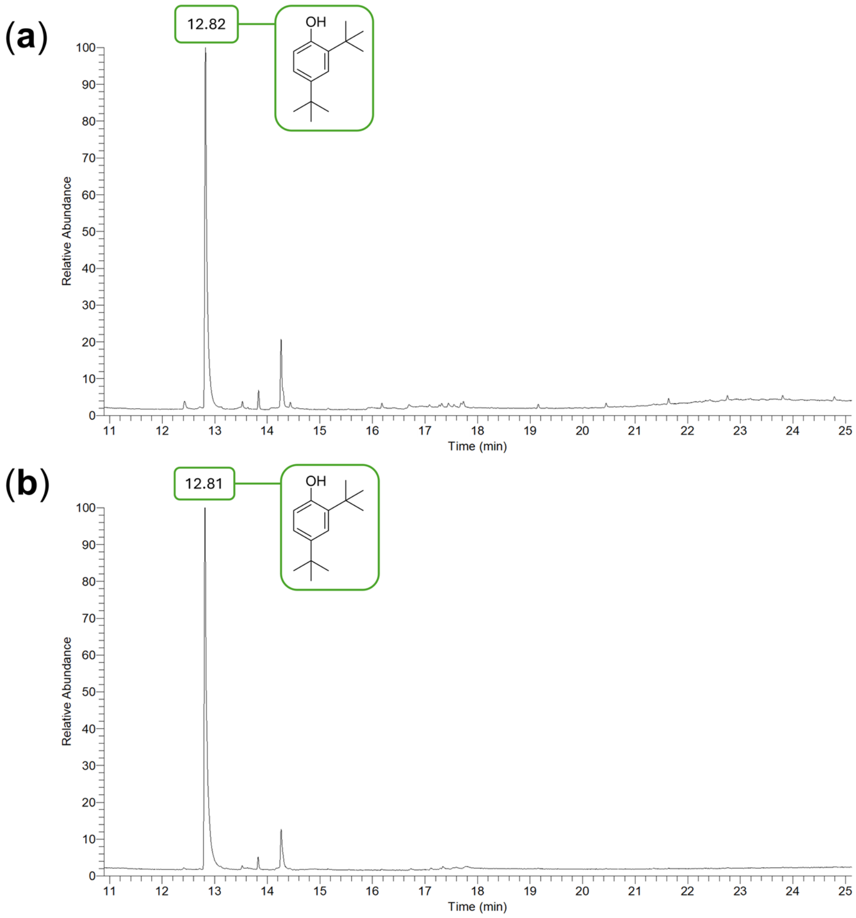

Figure S1. GC chromatograms for the THF solutions derived from reactions of (**a**) **DNIC-COOH** and (**b**) **DNIC-COOMe** with 10 equiv. of O_2 in the presence of 4 equiv. of DTBP at ambient temperature for overnight. In addition to the indicative peak of DTBP at RT = 12.8 min, the peaks for NO_2-DTBP at RT = ~15 min and coupled bisphenol at RT = ~21 min are not observed.[11]

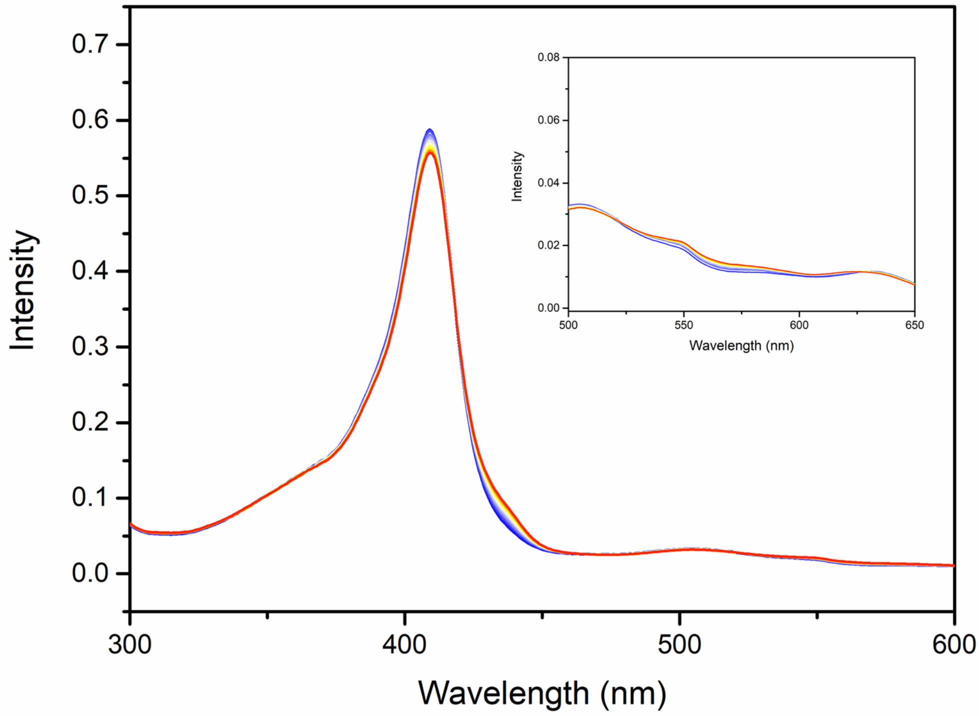

Figure S2. Reaction of metMb (5 μM) with Cys (200 μM) under anaerobic condition monitored by UV-vis spectroscopy. UV-vis spectra were measured every 0.5 h for a duration of 4.5 h.

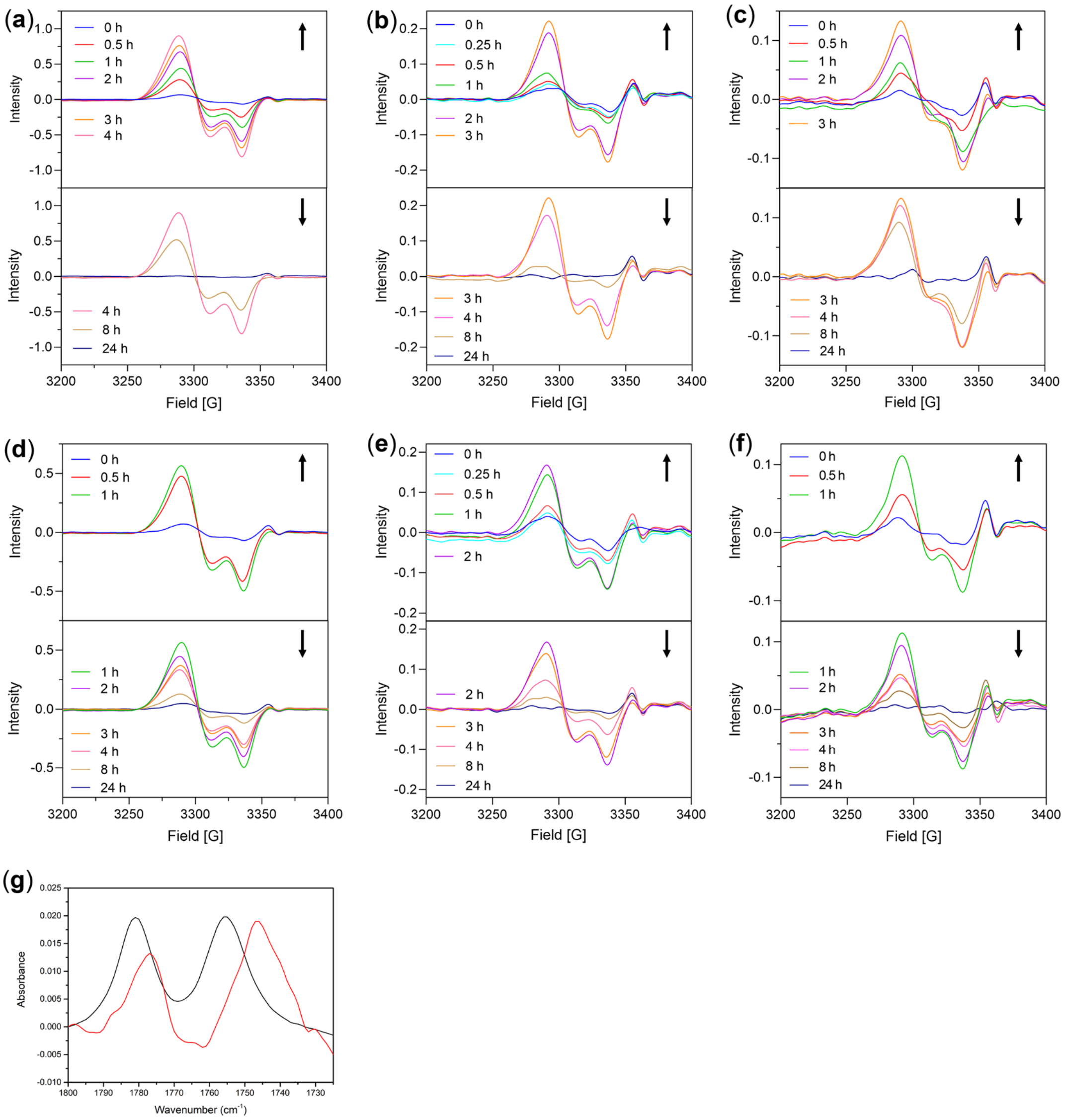

Figure S3. Time-dependent change of EPR spectra for **DNIC-COOH** (50 μM) in (**a**) αMEM with 20% FBS, (**b**) MEM with 5% FBS, and (**c**) HSFM with 2% FBS under normoxia conditions. Time-dependent change of EPR spectra for **DNIC-COOMe** (50 μM) in (**d**) αMEM with 20% FBS, (**e**) MEM with 5% FBS, and (**f**) HSFM with 2% FBS under normoxia conditions. (**g**) IR spectra for **DNIC-COOH** (500 μM) before (black) and after (red) reaction with BSA (50 mg/mL) in deuterated 25 mM KPi buffer (pH 7.4).

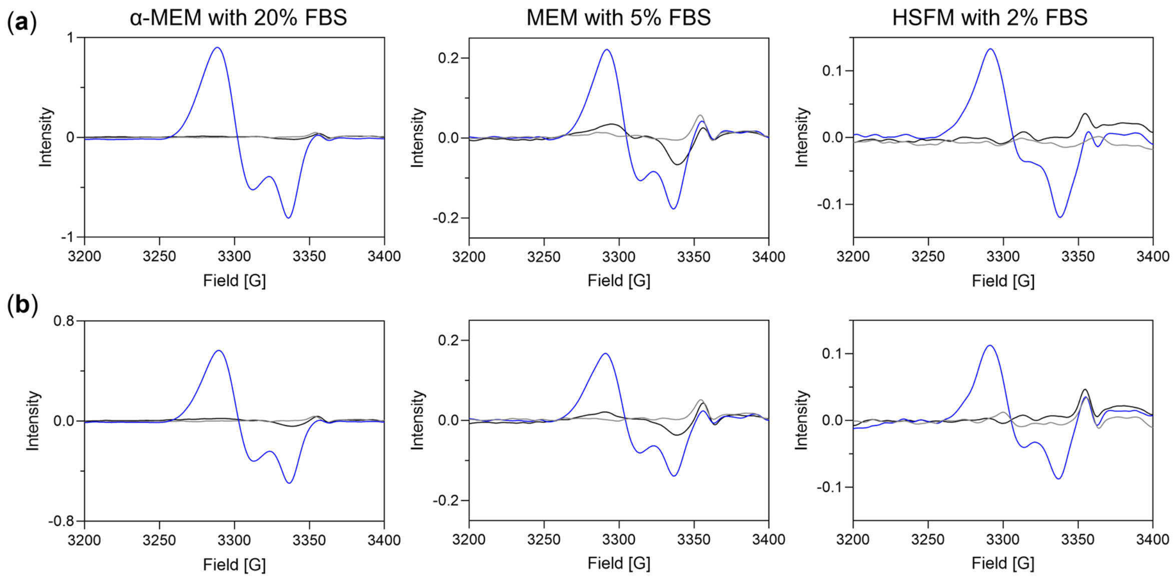

Figure S4. EPR spectra for (**a**) **DNIC-COOH** (gray, 50 μM) and (**b**) **DNIC-COOMe** (gray, 50 μM) under alternative cell culturing media without FBS. EPR spectra for (**a**) **DNIC-COOH** and (**b**) **DNIC-COOMe** under alternative cell culturing media with native FBS and NEM-treated FBS are depicted in blue and black, respectively.

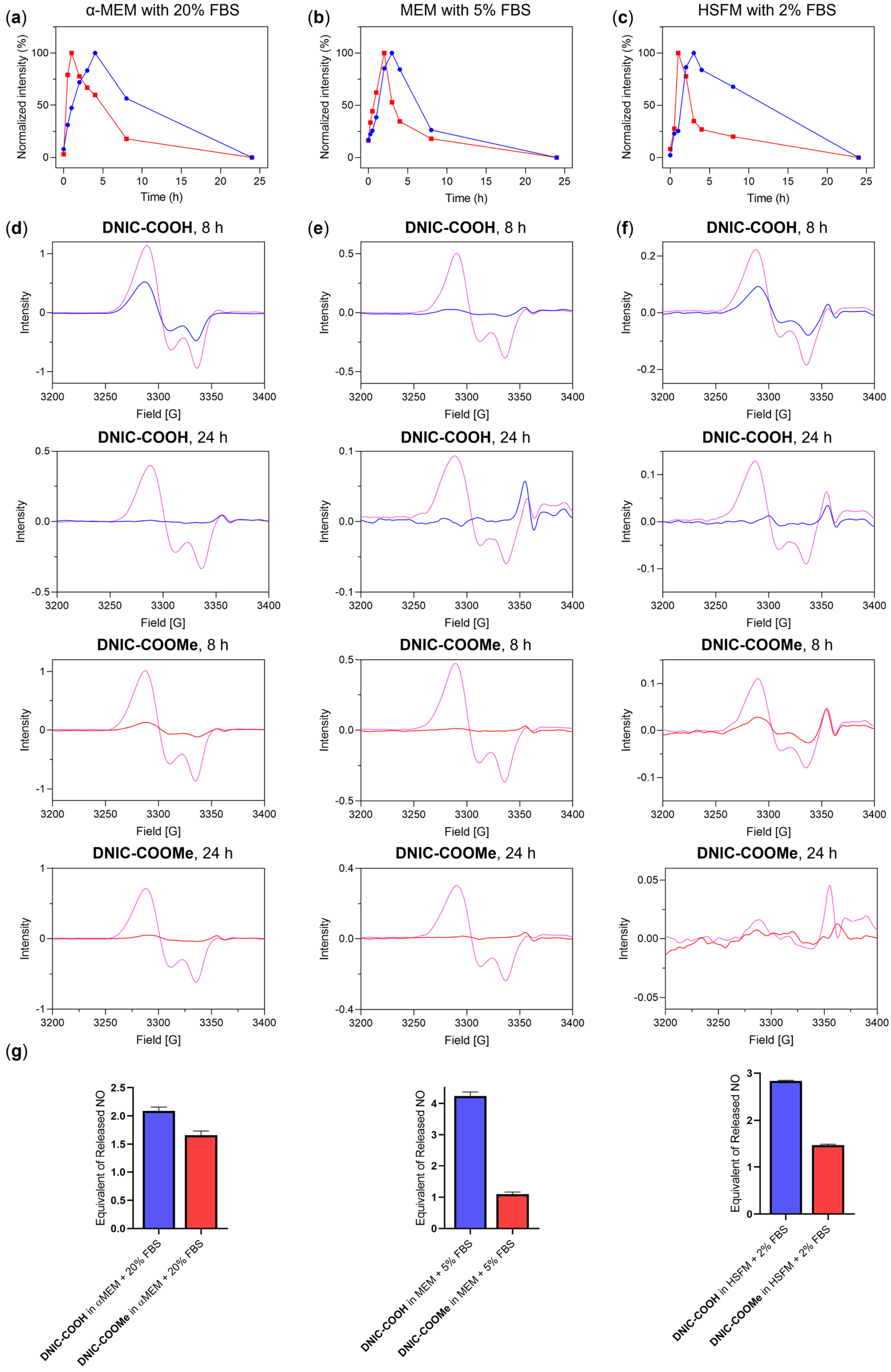

(a) α-MEM with 20% FBS
(b) MEM with 5% FBS
(c) HSFM with 2% FBS
Normalized intensity (%)
Time (h)
(d) (e) (f)
DNIC-COOH, 8 h
DNIC-COOH, 24 h
DNIC-COOMe, 8 h
DNIC-COOMe, 24 h
Intensity
Field [G]
(g)
Equivalent of Released NO
DNIC-COOH in αMEM + 20% FBS
DNIC-COOMe in αMEM + 20% FBS
DNIC-COOH in MEM + 5% FBS
DNIC-COOMe in MEM + 5% FBS
DNIC-COOH in HSFM + 2% FBS
DNIC-COOMe in HSFM + 2% FBS

Figure S5. Formation and decay of albumin-bound DNIC derived from the reaction of BSA and **DNIC-COOH** (50 μM, blue) or **DNIC-COOMe** (50 μM, red) in (**a**) αMEM with 20% FBS, (**b**) MEM with 5% FBS, and (**c**) HSFM with 2% FBS under normoxia condition. EPR spectra for **DNIC-COOH** (50 μM) or **DNIC-COOMe** (50 μM) incubated in (**d**) αMEM with 20% FBS, (**e**) MEM with 5% FBS, and (**f**) HSFM with 2% FBS under normoxia condition (blue for **DNIC-COOH** and red for **DNIC-COOMe**) or hypoxia condition (magenta) for 8 h and 24 h, respectively. (**g**) Equivalent of released NO during degradation of DNICs in alternative cell culturing media under normoxia condition. The data are the mean values ± SD pooled from three independent experiments.

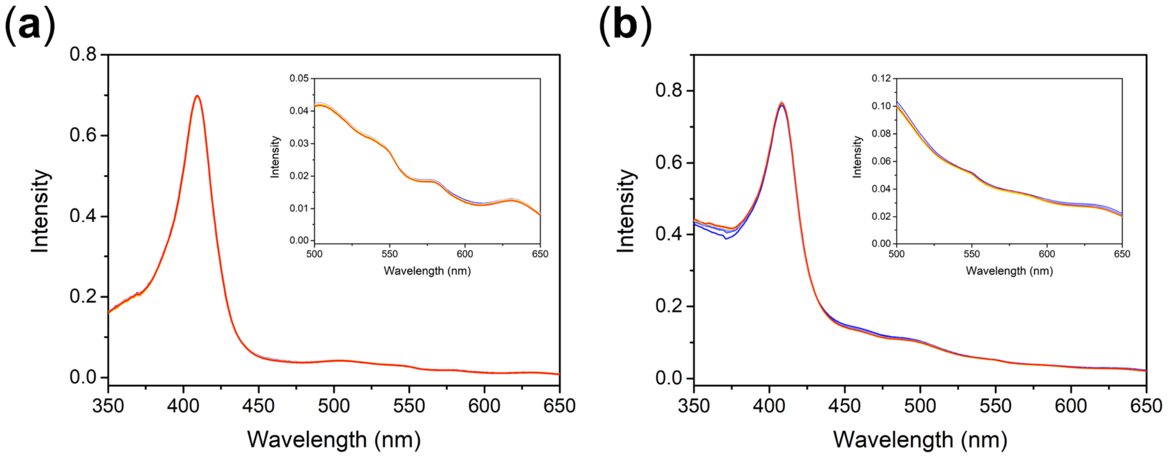

Figure S6. Reactions of metMb (5 μM) with (**e**) **DNIC-COOH** (5 μM) and BSA (50 mg/mL) and (**f**) **DNIC-COOMe** (5 μM) and BSA (50 mg/mL), respectively, under anaerobic condition monitored by UV-vis spectroscopy. UV-vis spectra were measured every 30 min.

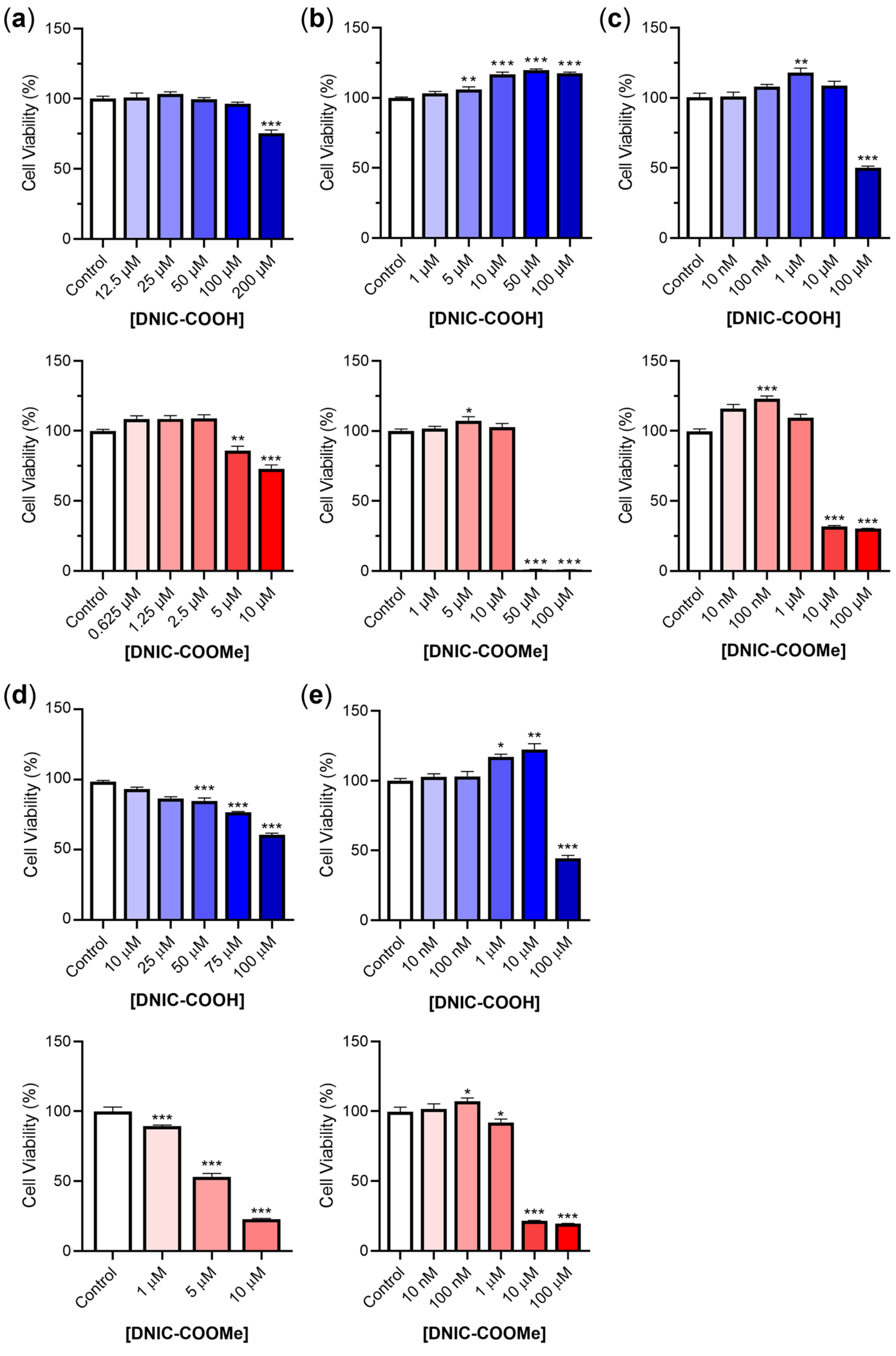

Figure S7. Cell viability of (**a**) MSC, (**b**) N2a, and (**c**) HCEC treated with different concentrations of **DNIC-COOH** and **DNIC-COOMe**, respectively, for 24 h. Data show the mean ± SEM (n = 3). *$p < 0.05$, **$p < 0.01$, and ***$p < 0.001$ in comparison with the control group. (**d**) Cell viability of HCEC treated with different concentrations of **DNIC-COOH** and **DNIC-COOMe**, respectively, for 24 h. ***$p < 0.001$ in comparison with the control group. (**e**) Cell viability of HCEC treated with different concentrations of **DNIC-COOH** and **DNIC-COOMe**, respectively, for 48 h. *$p < 0.05$, **$p < 0.01$, and ***$p < 0.001$ in comparison with the control group. Data show the mean ± SEM (n = 3).

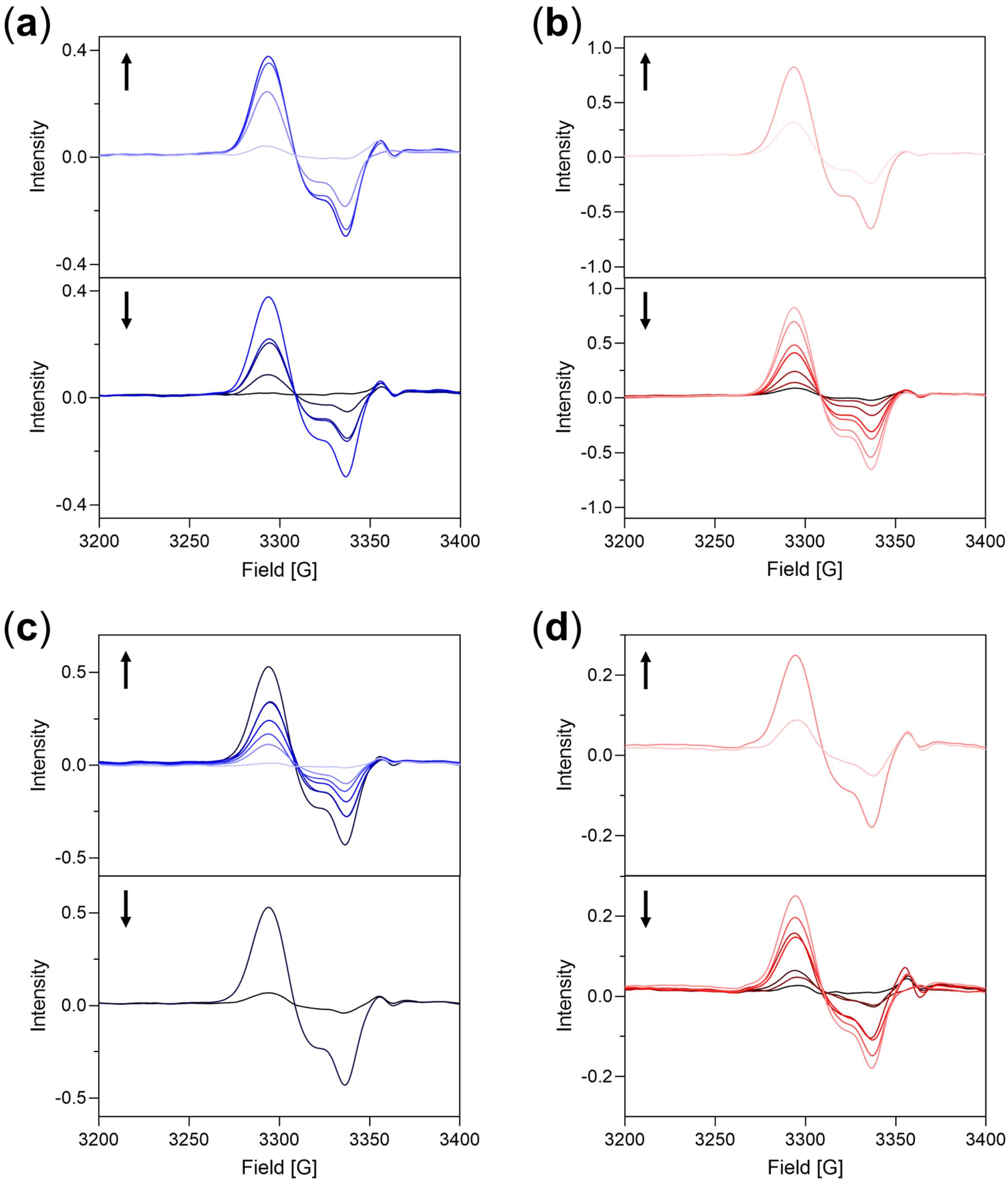

Figure S8. Time-dependent change of EPR spectra for (**a-b**) N2a and (**c-d**) HCEC treated with **DNIC-COOH** (blue) and **DNIC-COOMe** (red), respectively.

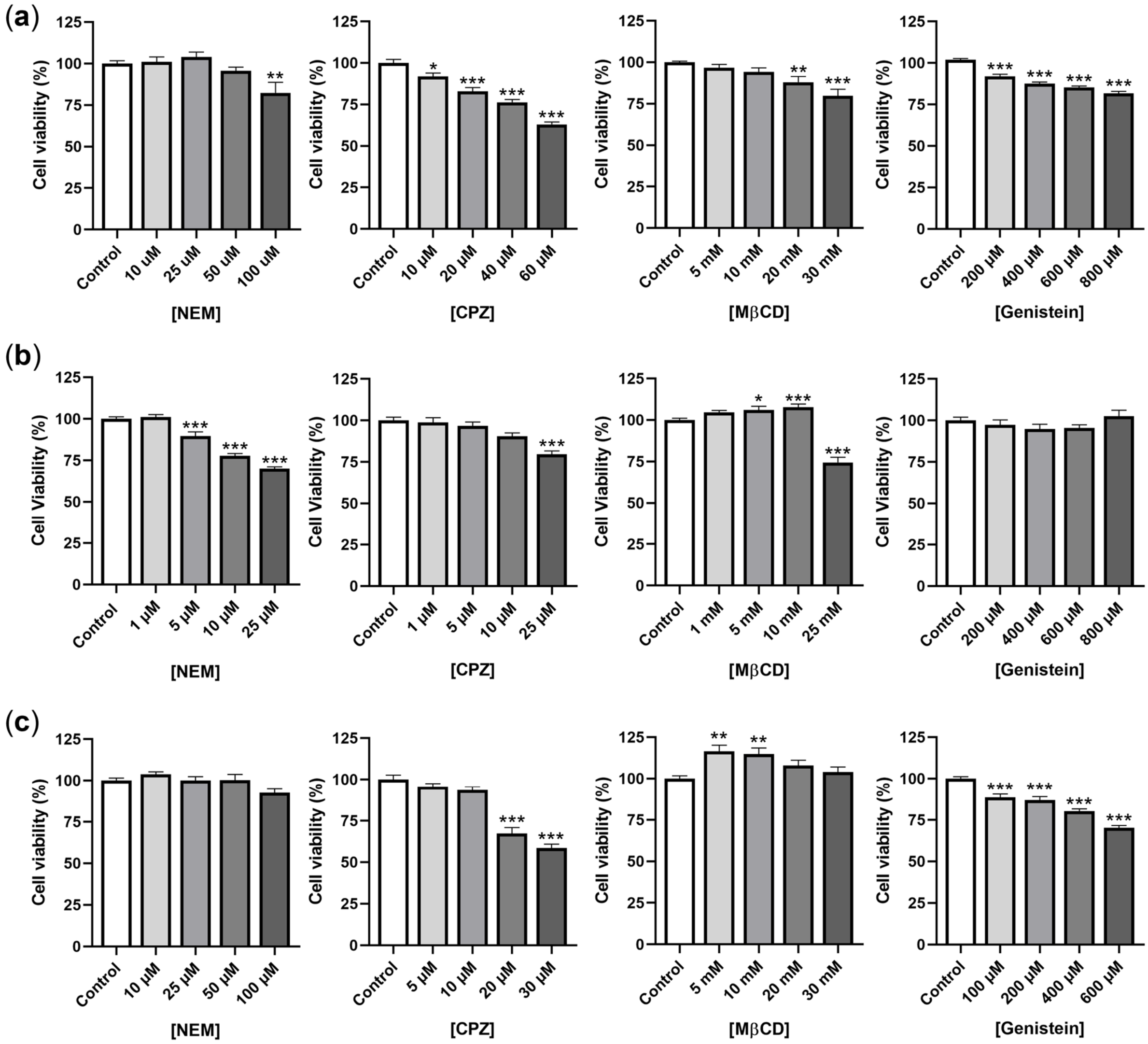

Figure S9. Cell viability of (**a**) MSC, (**b**) N2a, and (**c**) HCEC received treatments of different concentrations of alternative inhibitors. *$p < 0.05$, **$p < 0.01$, and ***$p < 0.001$ for comparison with the control group. Data show the mean ± SEM (n = 3).

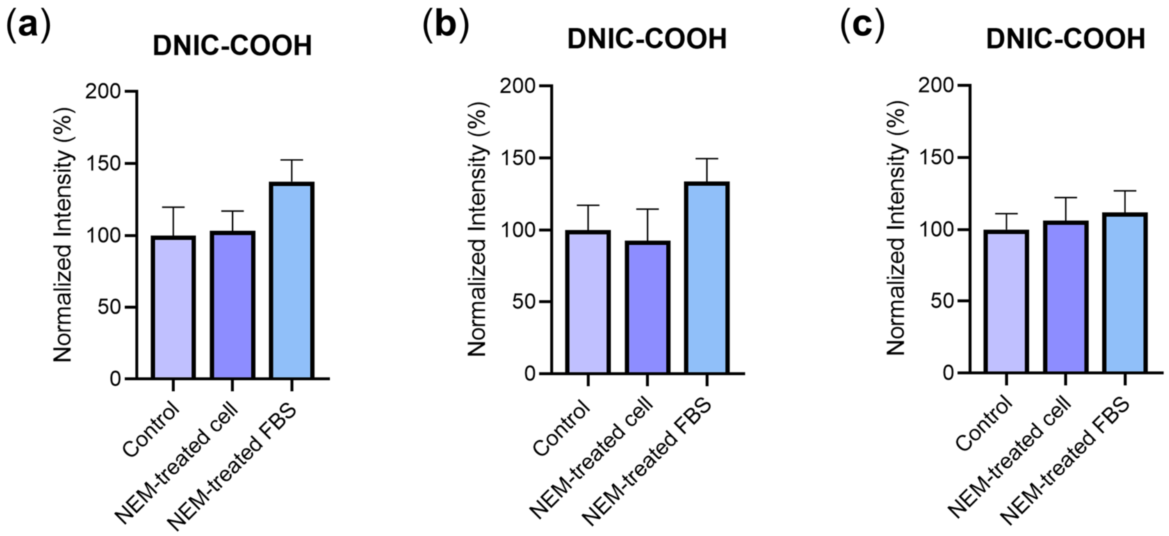

Figure S10. Relative EPR intensity at g_{av} = 2.03 for (**a**) MSC, (**b**) N2a, and (**c**) HCEC received sequential treatments of alternative inhibitors and **DNIC-COOH**. The cells received treatments of only **DNIC-COOH** (control group) are normalized as 100%. Data show the mean ± SEM (n = 3).

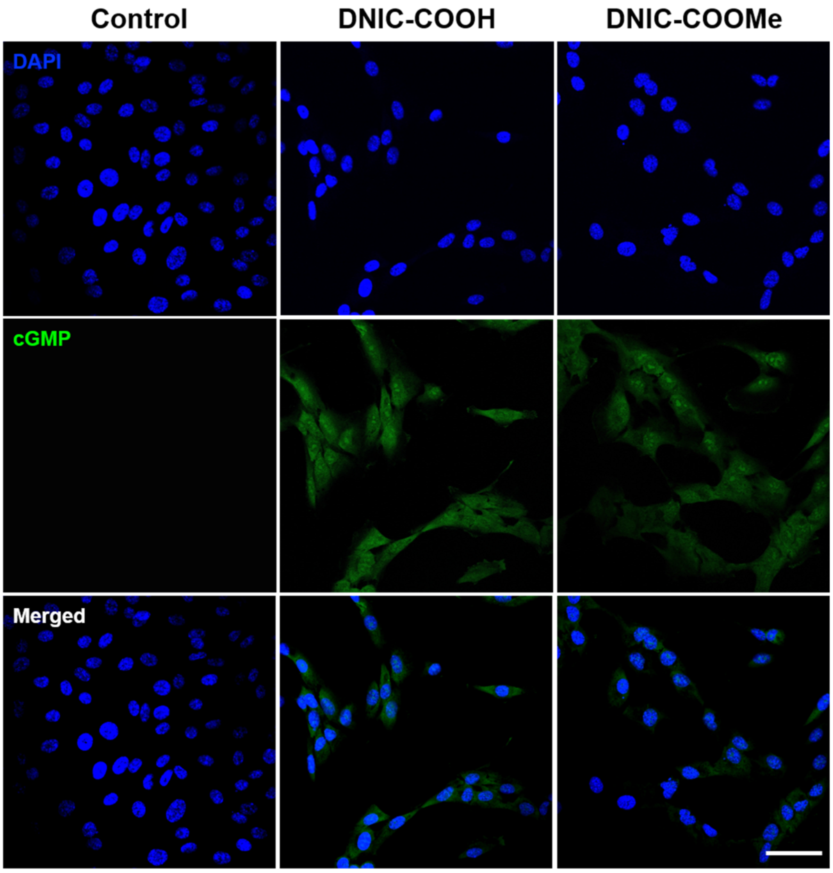

Figure S11. Confocal microscopic study on intracellular formation of cGMP in the MSC without (control group) or with the treatments of **DNIC-COOH** and **DNIC-COOMe**, respectively. Scale bar = 50 μm.

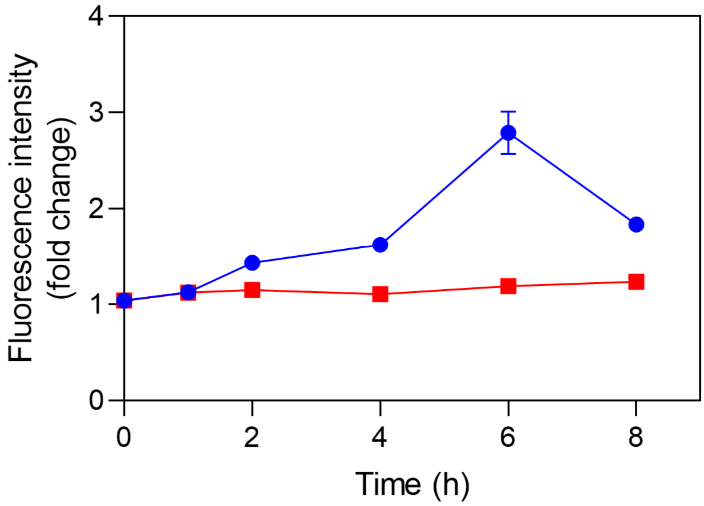

Figure S12. NO-release profile of **DNIC-COOH** without (blue) or with the presence of PTIO (red). Data show the mean ± SEM (n = 3).

Reference

1. Pokidova, O. V.; Novikova, V. O.; Emel'yanova, N. S.; Kormukhina, A. Y.; Kulikov, A. V.; Utenyshev, A. N.; Lazarenko, V. A.; Ovanesyan, N. S.; Starostina, A. A.; Sanina, N. A., A Nitrosyl Iron Complex with 3.4-Dichlorothiophenolyl Ligands: Synthesis, Structures and its Reactions with Targets - Carriers of Nitrogen Oxide (NO) *in vivo*. *Dalton Trans.* **2023,** *52* (9), 2641-2662.

2. Pokidova, O. V.; Emel'yanova, N. S.; Kormukhina, A. Y.; Novikova, V. O.; Kulikov, A. V.; Kotelnikov, A. I.; Sanina, N. A., Albumin as a Prospective Carrier of the Nitrosyl Iron Complex with Thiourea and Thiosulfate Ligands under Aerobic Conditions. *Dalton Trans.* **2022,** *51*, 6473-6485.

3. Pokidova, O.; Rudneva, T.; Tretyakov, B.; Kotelnikova, R.; Kotelnikov, A.; Aldoshin, S., Influence of Hemoglobin and Albumin on the NO Donation Effect of Tetranitrosyl Iron Complex with Thiosulfate. *Nitric Oxide* **2020,** *94*, 69-72.

4. Truzzi, D. R.; Alves, S. V.; Netto, L. E. S.; Augusto, O., The Peroxidatic Thiol of Peroxiredoxin 1 is Nitrosated by Nitrosoglutathione but Coordinates to the Dinitrosyl Iron Complex of Glutathione. *Antioxidants-Basel* **2020,** *9* (4), https://doi.org/10.3390/antiox9040276.

5. Turella, P.; Pedersen, J. Z.; Caccuri, A. M.; De Maria, F.; Mastroberardino, P.; Lo Bello, M.; Federici, G.; Ricci, G., Glutathione Transferase Superfamily Behaves Like Storage Proteins for Dinitrosyl-Diglutathionyl-Iron Complex in Heterogeneous Systems. *J. Biol. Chem.* **2003,** *278* (43), 42294-42299.

6. Wu, S.-C.; Lu, C.-Y.; Chen, Y.-L.; Lo, F.-C.; Wang, T.-Y.; Chen, Y.-J.; Yuan, S.-S.; Liaw, W.-F.; Wang, Y.-M., Water-Soluble Dinitrosyl Iron Complex (DNIC): a Nitric Oxide Vehicle Triggering Cancer Cell Death via Apoptosis. *Inorg. Chem.* **2016,** *55* (18), 9383-9392.

7. Vanin, A. F.; Pekshev, A. V.; Vagapov, A. B.; Sharapov, N. A.; Lakomkin, V. L.; Abramov, A. A.; Timoshin, A. A.; Kapelko, V. I., Gaseous Nitric Oxide and Dinitrosyl Iron Complexes with Thiol-Containing Ligands as Potential Medicines that Can Relieve COVID-19. *Biophysics* **2021,** *66* (1), 155-163.

8. Burgovsmall a, C. E. N.; Khristidis, Y. I.; Kurkov, A. V.; Mikoyan, V. D.; Shekhter, A. B.; Adamyan, L. V.; Timashev, P. S.; Vanin, A. F., The Inhibiting Effect of Dinitrosyl Iron Complexes with Thiol-containing Ligands on the Growth of Endometrioid Tumours in Rats with Experimental Endometriosis. *Cell. Biochem. Biophys.* **2019,** *77* (1), 69-77.

9. Truzzi, D. R.; Augusto, O.; Iretskii, A. V.; Ford, P. C., Dynamics of Dinitrosyl Iron Complex (DNIC) Formation with Low Molecular Weight Thiols. *Inorg. Chem.* **2019,** *58* (19), 13446-13456.

10. Pectol, D. C.; Khan, S.; Elsabahy, M.; Wooley, K. L.; Lim, S. M.; Darensbourg, M. Y., Effects of Glutathione and Histidine on NO Release from a Dimeric Dinitrosyl Iron Complex (DNIC). *Inorg. Chem.* **2020,** *59* (23), 16998-17008.

11. Liao, C.-J.; Tseng, Y.-T.; Cheng, Y.-A.; Dayao, L. A.; Iffland-Muhlhaus, L.; Gee, L. B.; Ribson, R. D.; Chan, T.-S.; Apfel, U.-P.; Lu, T.-T., Ligand Control of Dinitrosyl Iron Complexes for Selective Superoxide-Mediated Nitric Oxide Monooxygenation and Superoxide-Dioxygen Interconversion. *J. Am. Chem. Soc.* **2023,** *145* (37), 20389-20402.
